# USP18 is a significant driver of memory CD4 T-cell reduced viability caused by type I IFN signaling during primary HIV-1 infection

**DOI:** 10.1371/journal.ppat.1008060

**Published:** 2019-10-28

**Authors:** Xavier Dagenais-Lussier, Hamza Loucif, Hugo Cadorel, Juliette Blumberger, Stéphane Isnard, Mariana Gé Bego, Éric A. Cohen, Jean-Pierre Routy, Julien van Grevenynghe, for the Montreal Primary Infection Study Group

**Affiliations:** 1 Institut national de la recherche scientifique (INRS)-Institut Armand-Frappier, 531 boulevard des Prairies, Laval, QC, Canada; 2 Chronic Viral Illness Service and Division of Hematology, McGill University Health Centre, Glen site, Montréal, Québec, Canada; 3 Institut de recherches cliniques de Montréal (IRCM), Montréal, QC, Canada; 4 Department of Microbiology, Infectiology and Immunology, Université de Montréal, Montreal, QC, Canada; Vaccine Research Center, UNITED STATES

## Abstract

The loss of Memory CD4 T-cells (Mem) is a major hallmark of HIV-1 immuno-pathogenesis and occurs early during the first months of primary infection. A lot of effort has been put into understanding the molecular mechanisms behind this loss, yet they still have not been fully identified. In this study, we unveil the unreported role of USP18 in the deleterious effects of sustained type I IFN signaling on Mem, including HIV-1-specific CD4 T-cells. We find that interfering with IFN-I signaling pathway in infected patients, notably by targeting the interferon-stimulated gene USP18, resulted in reduced PTEN expression similar to those observed in uninfected control donors. We show that AKT activation in response to cytokine treatment, T-cell receptor (TcR) triggering, as well as HIV-1 Gag stimulation was significantly improved in infected patients when PTEN or USP18 were inhibited. Finally, our data demonstrate that higher USP18 in Mem from infected patients prevent proper cell survival and long-lasting maintenance in an AKT-dependent manner. Altogether, we establish a direct role for type I IFN/USP18 signaling in the maintenance of total and virus-specific Mem and provide a new mechanism for the reduced survival of these populations during primary HIV-1 infection.

## Author summary

In this study, we expend our knowledge of how type I interferons (IFN-I) leads to memory CD4 T-cell defective survival by unveiling the molecular mechanism behind such impairments, placing USP18 at its center. Our data further deciphers the specific USP18-related mechanism that is responsible for such impairments by implicating AKT inhibition in a PTEN-dependent manner. Our findings also point to a potential use of neutralizing anti-interferon α/β receptor antibodies to rescue the defective memory CD4 T-cell survival during HIV-1 infection, even in HIV-1 specific CD4 T-cell. To conclude, our findings provide the characterization of the molecular pathway leading to disturbances caused by sustained IFN-I signaling which occurs early during primary HIV-1 infection, complementing current knowledge which placed sustained IFN-I signaling as detrimental to the host during this infection.

## Introduction

The maintenance of memory CD4 T-cells (Mem) represents a key component for long-lasting immune protection during persistent infections with human immunodeficiency virus type 1 (HIV-1) and simian immunodeficiency virus (SIV) [1–4]. In this context, the elite controllers, who naturally control HIV-1 infection for decades in the absence of anti-retroviral therapy (ART), display the ability to maintain a large pool of Mem [4, 5]. For the other infected patients, loss of Mem occurs in the first months of HIV-1 infection, which exacerbates viral progression [6]. This loss depends on multiple molecular mechanisms, including metabolic disturbances, and is driven by prolonged inflammation alongside viral persistence [6–9]. Recently, our group identified a critical metabolic disturbance in the form of increased kynurenine levels in Mem from HIV-1-infected patients leading to higher production of reactive oxygen species and reducing cell responsiveness to IL-2 cytokine [6]. However, our knowledge of the different molecular mechanisms responsible for these immune defects remains incomplete as a full normalization of those defects is still unachieved. Therefore, we need to identify new factors responsible for these impairments and, more importantly, we need to establish an overall network regarding the relations between such impairments. Shedding light on such a network will enable better treatment, since individual factors are not enough to understand the full clinical picture of the disease.

Type I interferons (IFN-I) are necessary in establishing an efficient adaptive and acquired immune response, especially in acute viral infections, and are largely produced by plasmacytoid dendritic cells (pDC) following their stimulation [10, 11]. After binding the interferon_α/β_ receptor (IFNAR), IFN-I trigger the activation of interferon-stimulated genes (ISG) through the Janus kinase/STAT signaling pathway. These ISG include various intrinsic restriction factors, cytokines, chemokines, and co-stimulatory molecules [10, 12–14]. During acute viral infections, IFN-I expression is subject to negative regulation, which controls cytokine levels upon viral clearance [10]. However, in the case of persistent viral infections, such as HIV-1, sustained production of IFN-α is observed and is mainly driven by viremia and systemic inflammation [15–20]. Increasing evidence shows that the sustained IFN-I production during persistent viral infections can be detrimental for the host and directly participates in immune impairments [21–27]. Such impairments include the expression of inhibitory factors that reduce antiviral immunity, T-cell hyper activation and cell exhaustion as well as HIV-specific T-cell dysfunctions [17, 21, 22, 27–29]. However, it remains unknown to what extent and by which molecular mechanisms sustained IFN-I signaling affects the survival of Mem and contributes to the loss of this population during primary HIV-1 infection.

As such, we investigated whether sustained IFN-I signaling during the early and later stages of HIV-1 infection impairs Mem survival and by which mechanisms these perturbations might occur. Our data show that Mem from infected patients display increased expression of the ISG ubiquitin specific peptidase 18 (USP18), also known as UBP43. In the past two decades, several functions of USP18 have been discovered: this protein is not only an isopeptidase, but also a major regulator of IFN-I signaling [30]. Under specific circumstances, USP18 binds to IFNAR2, one the subunit of the IFNAR dimer, and compete with JAK preventing proper activation of the pathway [31]. Therefore, USP18 functions as a maestro of many biological pathways in various cell types. However, no information are available regarding the contribution of USP18 on HIV-1 immuno-pathogenesis, we investigated its impact on Mem survival and function in infected subjects. Our study identified a critical role of USP18 in the loss of Mem including HIV-1-specific cells during HIV-1 infection. Our findings also demonstrate our ability to rescue Mem from apoptosis in a PTEN- and AKT-dependent manner when USP18 is specifically targeted.

Altogether, this study puts USP18/PTEN/AKT at the center of the molecular pathway by which sustained IFN-I signaling leads to Mem impairments during HIV-1 infection.

## Results

### Mem from HIV-1-infected subjects display higher USP18 expression, which can be normalized by IFNAR blockade

**Fig 1:**
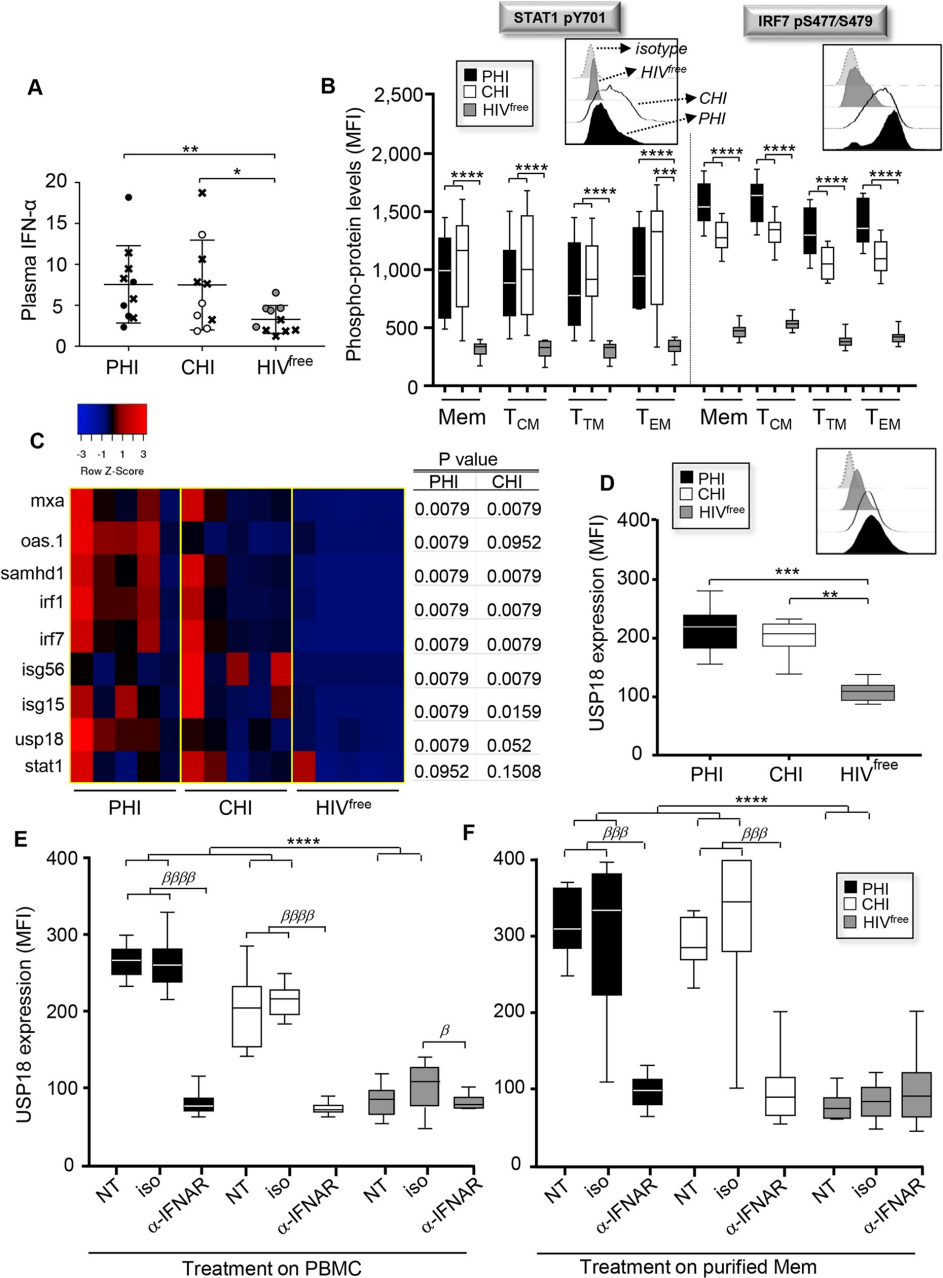
Mem from PHI and CHI display higher expression levels of USP18. (**A**) Plasma concentration of IFN-α in pg/mL for PHI, CHI and HIV^free^ subjects. Crosses represent subjects used in **C**. (n = 10). (**B**) Levels of STAT1 pY701 and IRF7 pS477/479 on 7-AAD^neg^CD3^+^CD4^+^CD45RA^neg^ Mem, as well as CD27^+^CCR7^+^ central memory (T_CM_), CD27^+^CCR7^neg^ transitional memory (T_TM_), and CD27^neg^CCR7^neg^ effector memory (T_EM_) subsets (MFI, mean fluorescence intensity) (n = 10). Representative histograms including isotype control are shown above. (**C)** Heat map representation of gene expression related to IFN-I signaling determined by real time RT-PCR on *ex vivo* Mem. The Z-score is the number of standard deviations from the mean data point. Reds are higher than the mean, blacks close to it and blues under it. Table shows *P* values of PHI or CHI compared to HIV^free^ (n = 5). (**D**) Expression of USP18 in *ex vivo* Mem from PHI, CHI and HIV^free^ (MFI) (n = 10). Representative histograms are also shown above. (**E,F**) Expression levels of USP18 in Mem after 24 hours of neutralizing α-IFNAR treatment (MFI) (n = 10). (**E**) α-IFNAR, its respective isotype control or no treatment (NT) have been administrated either on total PBMC or (**F**) directly on purified Mem. The error bars indicate standard deviations from the means. *β*, symbol used for paired *t* test (comparison between treated Mem and control). *, symbol used for Mann-Whitney test (comparison between study groups).

Despite indications of an IFN-I signature in HIV-1 infection [22, 32–34], the status of IFN-I signaling intrinsic to Mem and how it may impair cell survival in infected subjects are unknown. First, we compared the plasma levels of IFN-α from primary-infected (PHI) and chronically-infected (CHI) subjects to age-matched uninfected donor controls (HIV^free^). S1 Table summarizes the clinical and virological data for all selected PHI and CHI subjects including viral loads (VL) and CD4 counts. Similarly to others, we found higher IFN-α levels in plasma from HIV-1-infected subjects compared to uninfected controls (Fig 1A) [15–20]. We also found that the subjects with high plasma IFN-α levels were the ones with the highest VL (correlation between the two parameters: *P* = 0.0187, r = 0.5334; n = 19) (S1 Fig).

To assess the IFN-I signaling intrinsic to Mem, we next measured in PHI, CHI and HIV^free^ subjects the constitutive phosphorylation levels of STAT1 and IRF7, two IFN-I-induced transcription factors, in *ex vivo* Mem by PhosFlow. Here, by using a multicolor-parameter flow cytometric analysis as previously described, we investigated IFN-I signaling on all Mem subsets classified by three surface markers, CD45RA, CD27 and CCR7 [35, 36]. Total Mem were defined by a CD3^+^CD4^+^CD45RA^neg^ phenotype (S2A Fig). Our data showed increased constitutive levels for STAT1 pY701, STAT1 pS727 and IRF7 pS477/S479 in Mem from PHI and CHI subjects when compared to HIV^free^ donors (Fig 1B, S2B Fig and S3A Fig). The increased levels of the three phospho-proteins during HIV-1 infection were observed across all memory subsets that are determined by differential expression of CD27 and CCR7 markers. In this context, memory subsets include the long-lasting CD27^+^CCR7^+^ central memory CD4 T-cells (T_CM_) as well as CD27^neg^CCR7^neg^ effector memory CD4 T-cells (T_EM_) (Fig 1B and S3A Fig). Although CD45RA^+^ CD4 T-cells also displayed higher phospho-protein levels in HIV-1-infected subjects when compared to uninfected controls, their levels did not correlate with the cell frequencies unlike Mem (S2B and S2C Fig). Increased IFN-I signaling in Mem from infected subjects was further confirmed by assessing the mRNA expression of several ISGs such as restriction factors (MxA, OAS.1 and SAMHD1), transcription regulators (IRF1 and IRF7), ISG_15_, ISG_56_ and USP18 (Fig 1C). Of note, we found similar mRNA expression of STAT1 in Mem for all study groups (Fig 1C, last lane).

Considering the lack of literature surrounding USP18 expression during HIV-1 infection, we next compared its protein levels in the three groups of subjects. We found increased constitutive USP18 expression in Mem from PHI and CHI compared to HIV^free^ subjects (*P* = 0.0001 and *P* = 0.002, respectively for PHI and CHI subjects; n = 10 [MFI]) (Fig 1D). Percentages of USP18^+^ Mem were also higher in PHI and CHI subjects when compared to uninfected controls (36.2 ± 15.3 [PHI], 29.8 ± 12.3 [CHI] and 7.9 ± 4 [HIV^free^]; *P* < 0.0001 and *P* = 0.0014, respectively for PHI and CHI) (S4A Fig). We confirmed increased USP18 expression in purified Mem from PHI and CHI subjects when compared to HIV^free^ controls by western blot (S4B and S4C Fig). Unsurprisingly, antiviral therapy (ART), when administrated early during the first months of infection and for approximately 2.5 years, led to viral suppression alongside a full normalization of both plasma IFN-α levels and intrinsic USP18 expressions in Mem (S1 Table and S5A and S5B Fig). To assess whether increased USP18 expression levels in Mem from viremic subjects were associated with higher expression of IFN_α/β_ receptors (IFNAR), we looked at the surface levels of both IFNAR1 and IFNAR2 in PHI, CHI and HIV^free^ subjects. Not only our data did not show increased expression of IFNAR in infected subjects, but we even found reduced constitutive expression of IFNAR1 in PHI when compared to uninfected controls (S6A and S6B Fig). Similarly, increased USP18 expression levels during HIV-1 infection could not be explained by different ratios of Mem subsets as no significant differences were found between the study groups (S6C Fig). Finally, to test our ability to interfere with USP18 expression by blocking IFN-I signaling, peripheral blood mononuclear cells (PBMC) or purified Mem for all groups were cultured with neutralizing antibodies against the IFN_α/β_ receptor (α-IFNAR) or respective isotype Ig control for 24 hours before assessing USP18 levels. Our data showed that the α-IFNAR treatment normalized USP18 levels in Mem from PHI and CHI subjects (Fig 1E and 1F). Of note, the presence of Ig controls did not impact USP18 levels as compared to untreated Mem (Fig 1E and 1F).

Altogether, our results show increases of USP18 expression in Mem from HIV-1-infected subjects and confirm the efficacy of IFNAR blockade in normalizing their USP18 levels at 24 post-treatment in the range of those from uninfected controls.

### Higher USP18 expression in Mem from HIV-1-infected subjects prevents optimal AKT activation in response to cytokine stimulation in a PTEN-dependent manner

**Fig 2:**
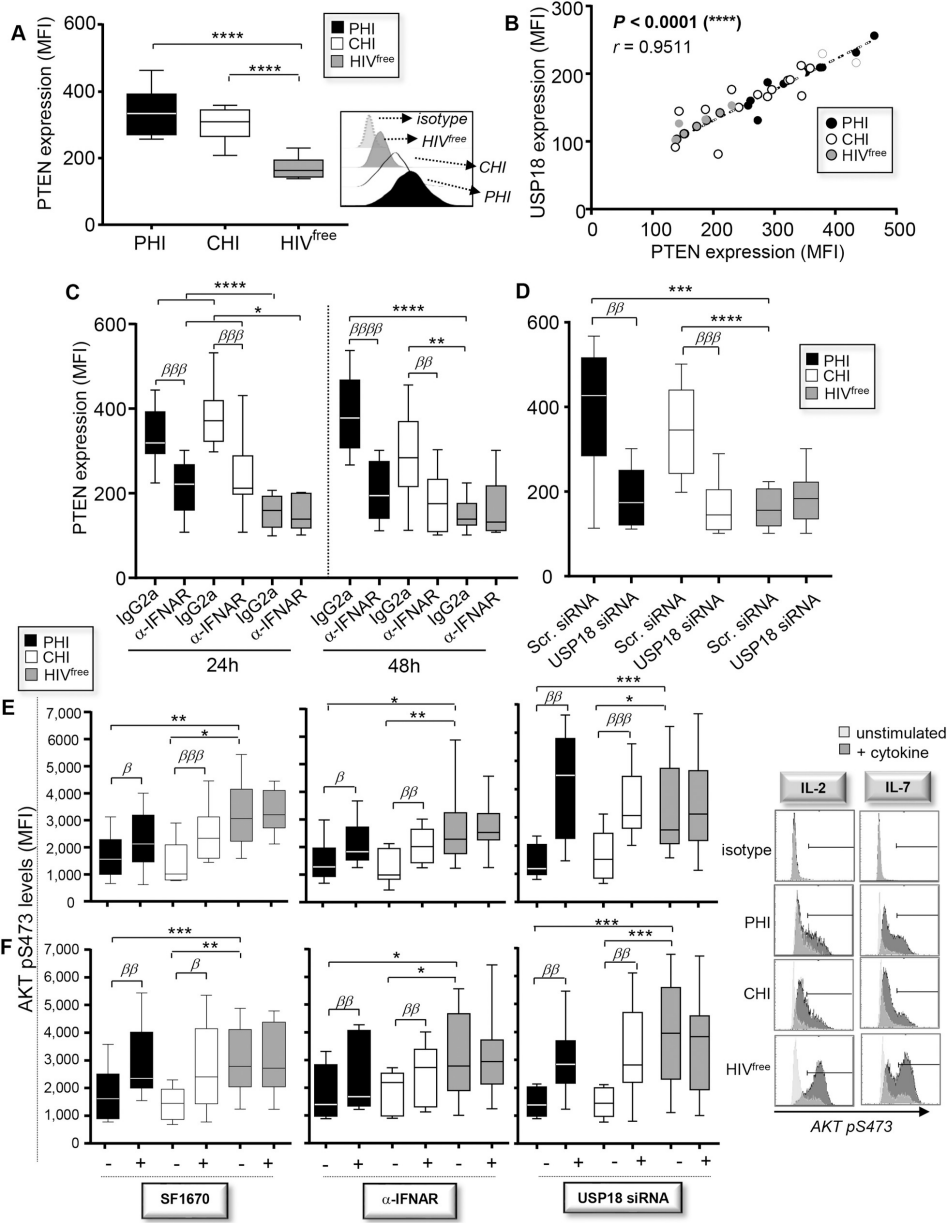
High USP18 expression in Mem from PHI and CHI impairs AKT activation. (**A**) *Ex vivo* PTEN expression levels in Mem from PHI, CHI and HIV^free^ subjects (MFI) (n = 10). Representative histograms including isotype control are also shown above. (**B**) Correlations between USP18 expression and PTEN expression in Mem for all subjects (n = 30). (**C**) PTEN expression levels in Mem that have been treated for 24 or 48 hours with α-IFNAR or its respective isotype control (n = 10). (**D**) Expression of PTEN in Mem that have been transfected with siRNA specific for USP18 or with scrambled siRNA (n = 10). (**E, F**) Levels of AKT pS473 following 15 minutes of IL-2 (**E**) or IL-7 (**F**) stimulation in Mem that have been pre-treated 48 hours with SF1670 (left), α-IFNAR or its respective isotype control (middle), or transfected or not for 48 hours with specific USP18 siRNA (right) (n = 10). Representative histograms for AKT p473 expression in cytokine-stimulated Mem for all groups of subjects including isotype control are also shown on the right side. The error bars indicate standard deviations from the means. *β*, symbol used for paired *t* test (comparison between treated Mem and control). *, symbol used for Mann-Whitney test (comparison between study groups).

Recently, data collected on lung cancer cell lines established USP18 as a potential regulator of PTEN protein levels and stability (41). To investigate whether higher USP18 expression may regulate PTEN expression in Mem during HIV-1 infection, we first assessed the constitutive *ex vivo* levels of PTEN in PHI, CHI, ART^+^ and HIV^free^ subjects. We found that PHI and CHI subjects displayed higher levels of PTEN compared to uninfected controls (*P* < 0.0001; n = 10 [MFI]) (Fig 2A). Similarly, the percentages of PTEN^+^ Mem were higher in PHI and CHI subjects when compared to HIV^free^ donors (63.3 ± 17.9 [PHI], 53.8 ± 16.2 [CHI] and 9.9 ± 7.3 [HIV^free^]; *P* < 0.0001) (S7A Fig). Of note, Mem from ART^+^ subjects displayed similar expression of PTEN than those from HIV^free^ controls (S5C Fig). We found highly significant correlation between USP18 and PTEN expression levels in Mem for all tested subjects (*P* < 0.0001, r = 0.9511; n = 30) (Fig 2B).

Next, we aimed to investigate whether interfering with USP18 levels impacts PTEN expression in Mem from HIV-1-infected subjects. PBMC from PHI, CHI and HIV^free^ subjects were first cultured with α-IFNAR or isotype control for 24 and 48 hours. Intracellular levels of PTEN were then determined on Mem by flow cytometry. Our data showed that a 24h-long treatment with α-IFNAR significantly reduced the levels of PTEN in PHI and CHI, yet it did not bring them down to the levels found in the HIV^free^ group (Fig 2C). By prolonging the α-IFNAR treatment to 48h, we found that the levels of PTEN in PHI and CHI were comparable to those found in HIV^free^ subjects. To further confirm that USP18 was the ISG responsible for regulating PTEN expression in Mem during HIV-1 infection, we specifically inhibited USP18 expression in Mem using small interfering RNAs (siRNA) silencing. Briefly, purified Mem from PHI, CHI and HIV^free^ subjects were either electroporated or transfected with siRNA specific for USP18 or with respective negative control siRNA for 2 hours, washed twice and then cultured with their autologous CD4-depleted PBMC (ratio Mem/PBMC = ¼). Both levels of USP18 and PTEN were finally determined by flow cytometry in transfected Mem for all groups at 48 hours post-transfection. Electroporation alone or transfection with negative siRNA did not affect USP18 expression when compared to Mem that were not electroporated. In contrast, Mem from infected subjects that were transfected with USP18 siRNA displayed an average of 79.4% and 82.4% reductions in USP18 expression, respectively for PHI and CHI subjects (S7B Fig). Same as for α-IFNAR treatments, USP18 silencing in Mem led to significant reductions of PTEN expression in PHI and CHI to levels comparable to the uninfected controls (Fig 2D). Of note, PTEN levels were similar between Mem that were electroporated alone and transfected with scrambled siRNA (S7C Fig).

Since PTEN is a negative regulator of PI3K/AKT signaling [37], we finally explored whether our HIV-1-infected groups had reduced AKT activation in response to IL-2 or IL-7 stimulations. We also assessed if we could rescue those activation levels by using either PTEN inhibitor SF1670, α-IFNAR or USP18 siRNA. In this context, we first pre-treated PBMC for all groups with or without SF1670, neutralizing α-IFNAR or its isotype control for 48 hours, and then stimulated the cells with cytokines for another round of 15 minutes before assessing AKT pS473 levels in Mem by PhosFlow. We also transfected purified Mem with USP18 siRNA or negative siRNA, cultured them with their autologous CD4-depleted PBMC for 48 hours and then added cytokines in cultures for 15 minutes before the FACS analysis. As expected, our data confirmed that Mem from PHI and CHI subjects displayed lower AKT activation in response to cytokine stimulations when compared to HIV^free^ controls (Fig 2E and 2F and S8A Fig). Basal levels of AKT pS473 determined in Mem that were not stimulated were similar between all tested groups (S8A and S8B Fig). Transcriptional analyses performed on purified Mem that have been stimulated or not with IL-2 or IL-7 showed similar mRNA expression of AKT except in the case of IL-7 stimulated Mem from CHI subjects (S3B Fig). Interestingly, our data showed that all pre-treatments, including USP18 silencing, led to significant improvements of cytokine-induced AKT activation in Mem from PHI and CHI in the range of uninfected controls (Fig 2E and 2F). Of note, levels of cytokine-induced AKT activations were similar between Mem that were electroporated alone and transfected with scrambled siRNA. Although we confirmed reduced IL-2-induced STAT5 pY694 levels in Mem from PHI and CHI subjects as previously reported [6], pre-treatments with α-IFNAR did not rescue STAT5 activation unlike AKT (S8C Fig). Finally, our data showed no significant differences for both basal and cytokine-induced AKT pS473 levels in Mem between ART^+^ subjects and uninfected controls (S5D Fig).

Overall, our data show that interfering with USP18 expression during HIV-1 infection leads to better AKT activation in a PTEN-dependent manner.

### Higher USP18 expression in infected subjects impairs Mem protection against Fas-induced apoptosis

**Fig 3:**
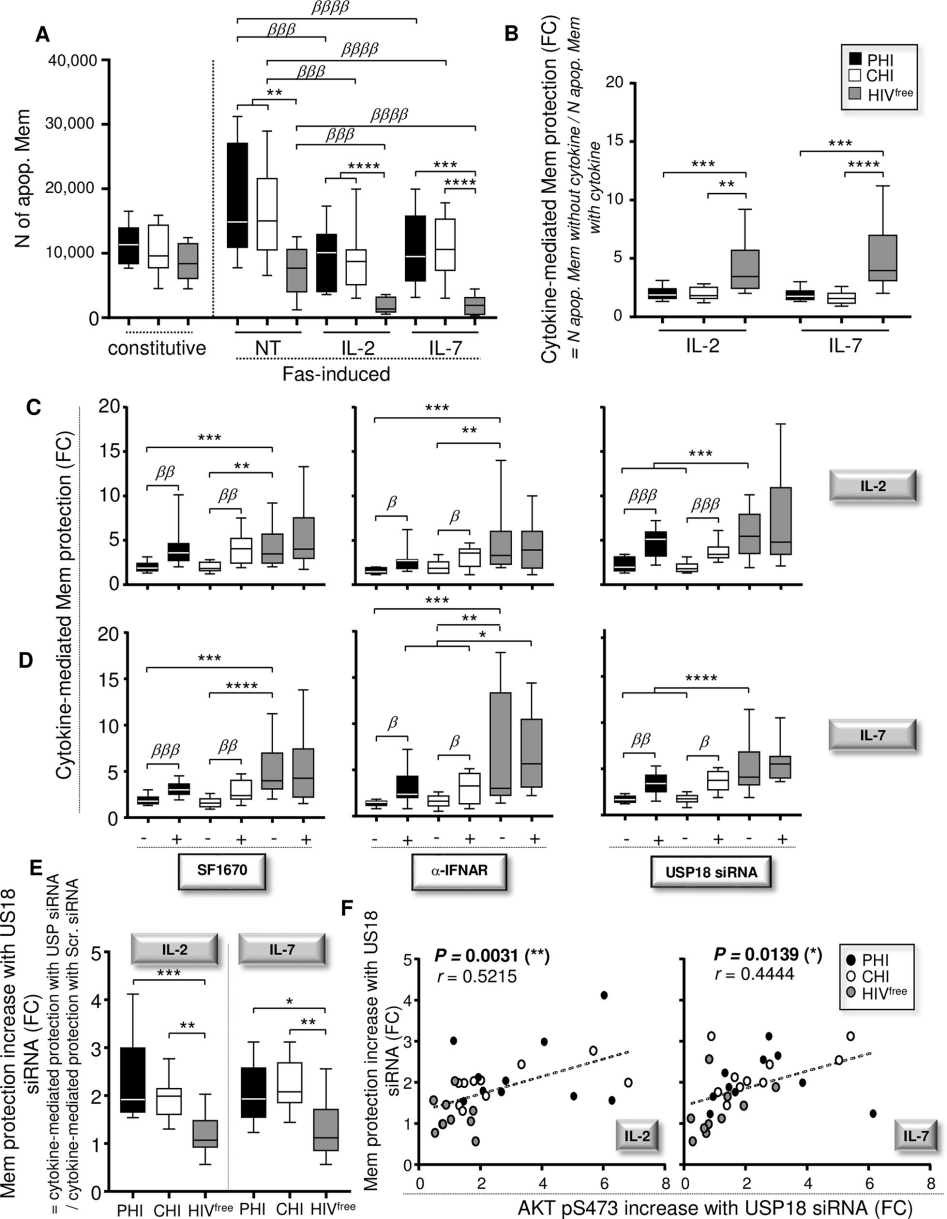
Interfering with USP18 in Mem from PHI and CHI improves cytokine responses and cell resistance to apoptosis. (**A**) Numbers (N) of constitutive apoptotic Mem (without any treatment) and Fas-induced apoptotic Mem in the presence or absence of IL-2 or IL-7 stimulation. N of Fas-induced apoptotic Mem were calculated according the formula: N of apoptotic Mem with CH11 –N of apoptotic Mem without CH11 (n = 10). (**B**) Cytokine-mediated Mem protection are shown in fold changes (FC). Cytokine-mediated Mem protection were determined by the formula: Number of Fas-induced apoptotic Mem without cytokine / Number of Fas-induced apoptotic Mem with cytokine (n = 10). (**C,D**) Fold changes of IL-2- (**C**) or IL-7 (**D**)-mediated Mem protection in cells that have been pre-treated for 48h with SF1670 (left), α-IFNAR or its respective isotype control (middle), or pre-transfected or not for 48 hours with USP18 siRNA (right) (n = 10). (**E**) Increases of Mem protection with USP18 siRNA determined in fold changes (FC). We calculated the increases of Mem protection in the context of USP18 targeting with the formula: cytokine-mediated Mem protection with USP18 siRNA / cytokine-mediated protection with scrambled siRNA. (**F**) Correlations between the increases of Mem protection and AKT activation after USP18 siRNA transfection in both IL-2- and IL-7-stimulated Mem (FC, fold change; n = 30). The error bars indicate standard deviations from the means. *β*, symbol used for paired *t* test (comparison between treated Mem and control). *, symbol used for Mann-Whitney test (comparison between study groups).

Since IL-2 and IL-7 play a critical role in regulating Mem survival [6, 38, 39], we next investigated their efficacy to protect Mem from Fas-induced apoptosis. To trigger Fas-induced apoptosis, we used the anti-Fas antibody (clone CH11), which activates the Fas signaling pathway in cultured cells [4, 6]. Briefly, PBMC from PHI, CHI and HIV^free^ subjects were treated or not for 24 hours with CH11 antibody in the presence or absence of IL-2 or IL-7 stimulations. At 24 hours of culture, we assessed the numbers of apoptotic cells and apoptosis levels in Mem using Annexin-V staining for all conditions (for the constitutive or basal, and Fas-induced apoptosis). We calculated the numbers (N) of Fas-induced apoptotic Mem as determined by the formula: N of apoptotic Mem with CH11—N of apoptotic Mem without CH11. Although we found a trend to higher numbers of Annexin-V^+^ Mem for the constitutive apoptosis in PHI and CHI when compared to HIV^free^ controls (10,020 ± 2,473; 10,484 ± 3,785; and 7,865 ± 3,785, respectively), our data show no significant differences between the study groups (Fig 3A, left side). However, in the absence of cytokine stimulation, we found significant higher numbers of Fas-induced apoptotic Mem from PHI and CHI subjects when compared to HIV^free^ subjects (*P* < 0.0001) (Fig 3A [right side] and S9A Fig). Similar results were found when using percentages of apoptosis instead of absolute numbers of Mem (S9B Fig). We found that the stimulations with IL-2 and IL-7 led to lower numbers of Fas-induced apoptotic Mem for all tested groups. In the same sets of experiments, we further decided to evaluate the levels of Mem protection against Fas-induced apoptosis when the cells are stimulated by cytokines. The levels of Mem protection with cytokine stimulation in fold changes (FC) were determined by the formula: N of Fas-induced apoptotic Mem without cytokine / N of Fas-induced apoptotic Mem with cytokine. We found that the levels of cytokine-mediated Mem protection were significantly lower in PHI and CHI subjects when compared to HIV^free^ controls (2.0 ± 0.5 [PHI], 2.1 ± 0.6 [CHI] and 4.3 ± 2.4 [HIV^free^]; 1.9 ± 0.5 [PHI], 1.6 ± 0.5 [CHI] and 4.9 ± 2.8 [HIV^free^], respectively for IL-2 and IL-7 stimulations; *P* ≤ 0.0016) (Fig 3B).

We next investigated whether cell pre-treatments with SF1670, α-IFNAR, or USP18 siRNA improve cytokine-mediated Mem protection in PHI and CHI subjects. Briefly, PBMC were pre-treated with or without SF1670, α-IFNAR or isotype control for 48 hours. As described below, pre-treated cells were then cultured for 24 hours in the presence or absence of CH11 antibodies and cytokines. Similarly, purified Mem were transfected with USP18 or scrambled siRNA and then cultured with their autologous CD4-depleted PBMC for 48 hours before assessing cytokine-mediated Mem protection. Not only did all pre-treatments significantly increased cytokine-mediated Mem protection in HIV-1-infected subjects, but specific USP18 silencing also brought the cell protection to levels comparable to the HIV^free^ group (Fig 3C and 3D). We found that the increased Mem resistance to apoptosis driven by USP18 gene silencing was not associated with a reduction of Fas receptor expression (S10 Fig). Finally, we calculated the increases of cytokine-mediated Mem protection in the context of USP18 silencing for all tested groups. The increases determined in fold changes (FC) were obtained using the formula: cytokine-mediated Mem protection with USP18 siRNA / cytokine-mediated Mem protection with scrambled siRNA. Our data showed that targeting USP18 expression in PHI and CHI subjects led to significant increases of cytokine-mediated Mem protection (Fig 3E). We found highly significant correlations between the increases of Mem protection against Fas-induced apoptosis and AKT activation in both IL-2- and IL-7-stimulated Mem in the context of USP18 gene silencing (*P* = 0.0031, r = 0.5215 and *P* = 0.0139, r = 0.4444 respectively; n = 30) (Fig 3F).

In summary, we show that interfering with USP18 during primary HIV-1 infection protects Mem from apoptosis in a PTEN and AKT-dependent manner and decreases the numbers of apoptotic Mem.

### Interfering with USP18 expression in HIV-1-infected subjects improves long-lasting Mem maintenance in an AKT-dependent manner

**Fig 4:**
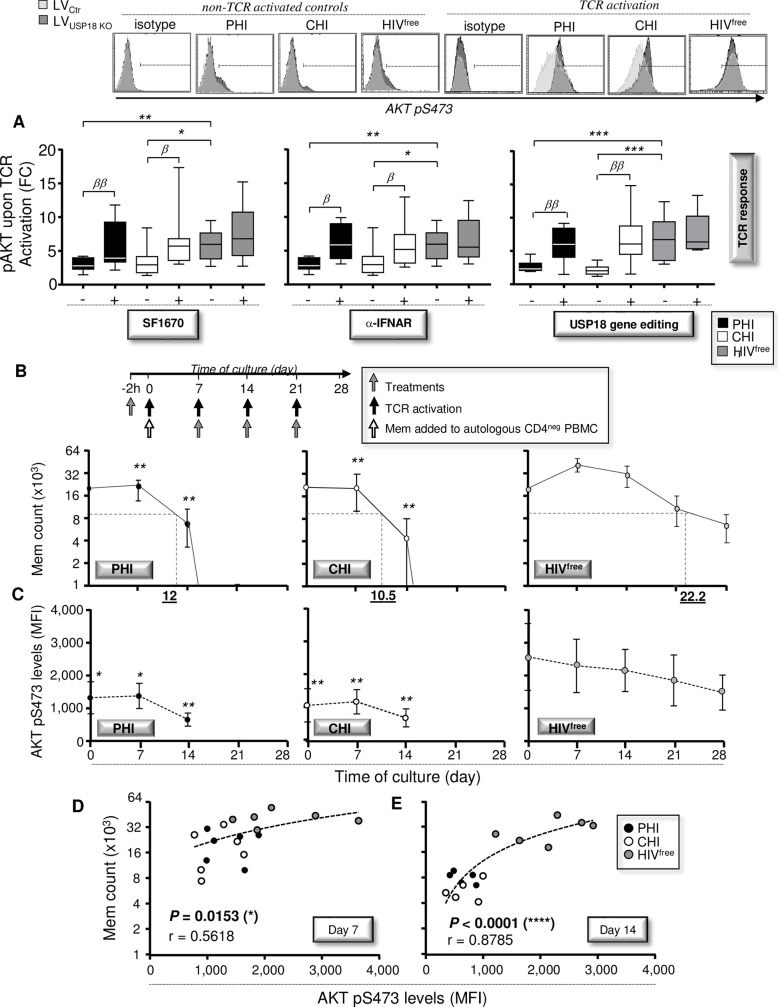
Reduced pAKT S473 after TcR triggering in PHI and CHI affects long-lasting Mem maintenance. (**A**) Levels of AKT pS473 following TcR activation for 30 minutes in Mem that have been pre-treated for 48h with SF1670 (left), α-IFNAR or its respective isotype control (middle). Levels of AKT pS473 in response to a 30 minute-long TcR activation have also been assessed in Mem that have been pre-transduced or not with LV_USP18 KO_ for 48 hours (right) (n = 10). Representative histograms for AKT pS473 expression for all conditions including the non-TCR activated and isotype controls are also shown in the upper side. (**B**) Mem counts from PHI, CHI, and HIV^free^ following TcR activation every 7 days for 28 days. Results are expressed as the average of six independent experiments ± SD in log_2_ scale. Dashed lines represent Mem half-lives for each group of subjects. (**C**) Levels of AKT pS473 following TcR activation every 7 days for 28 days. (**D,E**) Correlations between cell counts and AKT pS473 levels in Mem at 7 (**D**) and 14 (**E**) days of culture (FC, fold change; n = 18). The error bars indicate standard deviations from the means. *β*, symbol used for paired *t* test (comparison between treated Mem and control). *, symbol used for Mann-Whitney test (comparison between study groups).

Since the TcR triggering results in rapid activation of PI3K/AKT signaling [39, 40], we decided to investigate if this activation was lower in Mem from the PHI and CHI groups compared to the HIV^free^ group and if interfering with PTEN, IFN-I signaling or specifically with USP18 would rescue this activation. First, PBMC from the three study groups where pre-treated or not with SF1670, α-IFNAR or isotype control for 48 hours, before being stimulated with anti-CD3 and anti-CD28 antibodies (Abs) for an additional 15 minutes. Levels of AKT pS473 were finally determined in activated Mem by PhosFlow for all conditions of cultures. We also activated purified Mem with anti-CD3 and anti-CD28 Abs, transduced them with lentiviral CRISPR/Cas9 vectors mediating USP18 gene editing (lentiviral vectors for USP18 knock-out; LV_USP18 KO_) or control lentiviral vectors (LV_Ctr_) for 4 hours, washed them twice and cultured them for 48 hours with their autologous CD4-depleted PBMC. At 48 hours post-transduction, cells were subjected to another 15-minute-long round of TcR activation before assessing AKT activation levels. Although the basal levels of AKT pS473 were similar in un-activated Mem from all tested groups, our data showed that AKT activation in response to TcR triggering was systematically lower in Mem from PHI and CHI subjects when compared to HIV^free^ donors (Fig 4A). Both pre-treatments of Mem with SF1670 or α-IFNAR led to significant improvements of AKT activation in response to TcR triggering to levels comparable to those in the HIV^free^ group (Fig 4A, left and middle panels). We found that a 48 hour-long Mem transduction with lentiviral CRISPR/Cas9 vectors mediated USP18 gene editing resulted in more than 87% inhibition of protein levels (S11A Fig). Of note, Mem that were transduced with control lentiviral vectors displayed similar USP18 expression when compared to uninfected Mem. Similarly to cell pre-treatments, we found that specifically interfering with USP18 expression in activated Mem led to significant improvements of AKT activation to levels comparable to those of the HIV^free^ group (Fig 4A, right panels).

Considering that Mem are first and foremost defined by their long-lasting maintenance, we next assessed the ability of Mem from PHI, CHI, and HIV^free^ subjects to persist up to 28 days of culture in response to multiple rounds of TcR triggering as previously done [4, 41]. Briefly, purified Mem were first stimulated with anti-CD3 and anti-CD28 antibodies in the presence or absence of SF1670, α-IFNAR or isotype control for 2 hours, washed twice and then cultured with their autologous CD4-depleted PBMC. Cultured cells were then re-stimulated at day 7, 14 and 21 of culture with or without the specific inhibitors. Once again, to specifically interfere with USP18 expression, we purified Mem at day 0 of cultures, transduced them with LV_USP18 KO_ or LV_Ctr_ for 4 hours, washed them twice and cultured them with their autologous CD4-depleted PBMC for 7 days. Cells were then re-stimulated at day 7, 14 and 21 of cultures. At day 7, 14, 21 and 28, total numbers of viable Mem were counted, and the half-lives of these cells were estimated for each study groups. We also determined in Mem the levels of apoptosis using Annexin-V staining and the expressions of USP18 and AKT pS473 by flow cytometry. We also assessed by ELISA the levels of both IFN-α and virus productions in supernatants during the time course of culture.

As expected, USP18 gene editing led to sustained inhibition of the elevated levels of USP18 expression in Mem from PHI and CHI subjects (S11B Fig). The elevated USP18 expression in Mem from viremic subjects could be explained by sustained IFN-α production during the time course of culture (S12A Fig). Our data showed that Mem from PHI and CHI subjects were not able to persist in culture, unlike Mem from HIV^free^ donors (Fig 4B). Total counts of viable Mem from HIV^free^ subjects were approximately 2.1-fold higher at day 7 and 5.3-fold higher at day 14 than those obtained from HIV-1-infected subjects (*P* ≤ 0.0043; n = 6). Strikingly, almost all Mem from HIV-1-infected subjects died by day 21, whereas Mem from uninfected controls continued to survive for at least 28 days after four rounds of activation. The half-lives of gated Mem obtained from PHI, CHI and HIV^free^ subjects were 12, 10.5, and 22.2 days, respectively (Fig 4B). Interestingly, the reduced ability of Mem from infected subjects to persist was associated with lower expression levels of AKT pS473 during the time course of culture (Fig 4C). In addition, we found positive correlations between the counts of viable Mem and expression levels of AKT pS473 at day 7 and day 14 of cultures (*P* = 0.0153, r = 0.5618 and *P* < 0.0001, r = 0.8785 respectively; n = 18) (Fig 4D and 4E).

**Fig 5:**
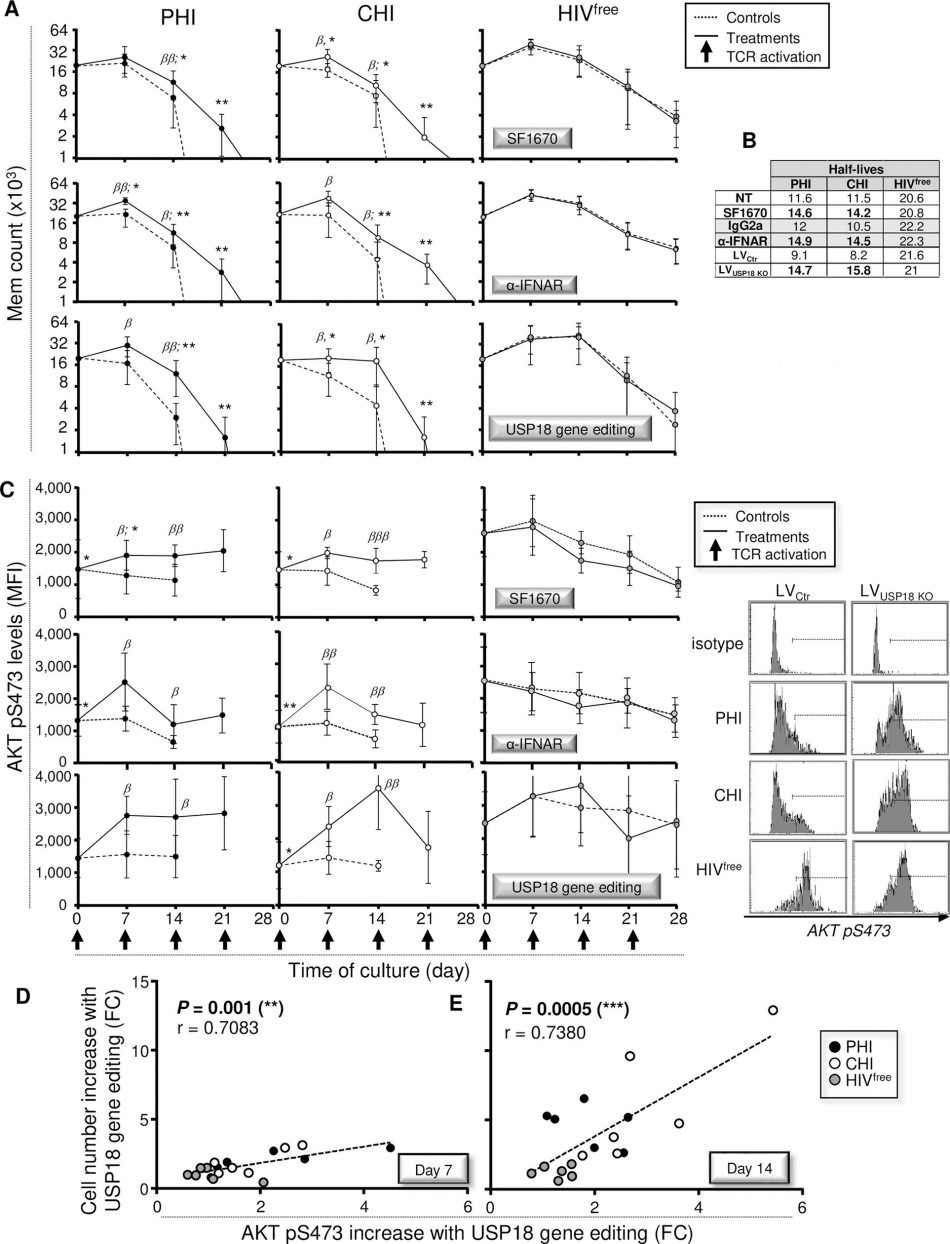
Targeting USP18 in PHI and CHI results in improved Mem maintenance in an AKT-dependent manner. (**A**) Mem counts from PHI, CHI, and HIV^free^ following TcR activation every 7 days for 28 days with or without SF1670 (top), α-IFNAR or its respective isotype control (middle), or CRISPR/Cas9 mediated USP18 gene editing (bottom) treatments. Results are expressed as the average of six independent experiments ± SD in log_2_ scale. (**B**) Half-lives were also determined for all study groups of subjects in the presence or absence of specific treatments. (**C**) Levels of AKT pS473 following TcR activation every 7 days for 28 days with or without SF1670 (top), α-IFNAR (middle), or CRISPR/Cas9 mediated USP18 gene editing (bottom) treatments. Representative histograms are also shown on the right side. (**D,E**) Correlations between the increases of cell counts and AKT pS473 levels after CRISPR/Cas9 mediated USP18 gene editing in Mem at 7 (**D**) and 14 (**E**) days of treatment (FC, fold change; n = 18). The error bars indicate standard deviations from the means. *β*, symbol used for paired *t* test (comparison between treated Mem and control). *, symbol used for Mann-Whitney test (comparison between study groups).

Treatments with SF1670, α-IFNAR or USP18 gene editing resulted in significant improvement of cell counts and half-lives in Mem from infected subjects from day 7 of culture (Fig 5A and 5B). Although all treatments significantly increased the numbers of viable Mem in PHI and CHI subjects during the cultures and allowed even some Mem to persist up to 21 days, they did not reach Mem counts found in cultures from HIV^free^ controls. In addition, although we systematically found HIV-1 production in cultures from infected subjects, USP18 interference did not impact the viral production (S12B Fig). This seemed to indicate that improvements of Mem survival, especially when USP18 was targeted, were not associated with reduced viral production in cultures. In contrast, all treatments led to significant increases of AKT pS473 levels intrinsic to Mem from PHI and CHI subjects (Fig 5C). We also found significant correlations between the increases of cell counts and AKT pS473 levels with USP KO in Mem at 7 and 14 days of treatment (*P* = 0.001, r = 0.7083 and *P* = 0.0005, r = 0.7380 respectively; n = 18) (Fig 5D and 5E).

Overall, our data confirm the efficacy of PTEN and USP18 interferences in improving long-lasting Mem maintenance in an AKT-dependent manner.

### Defective long-lasting Mem maintenance in HIV-1-infected subjects is explained by increased cell death

**Fig 6:**
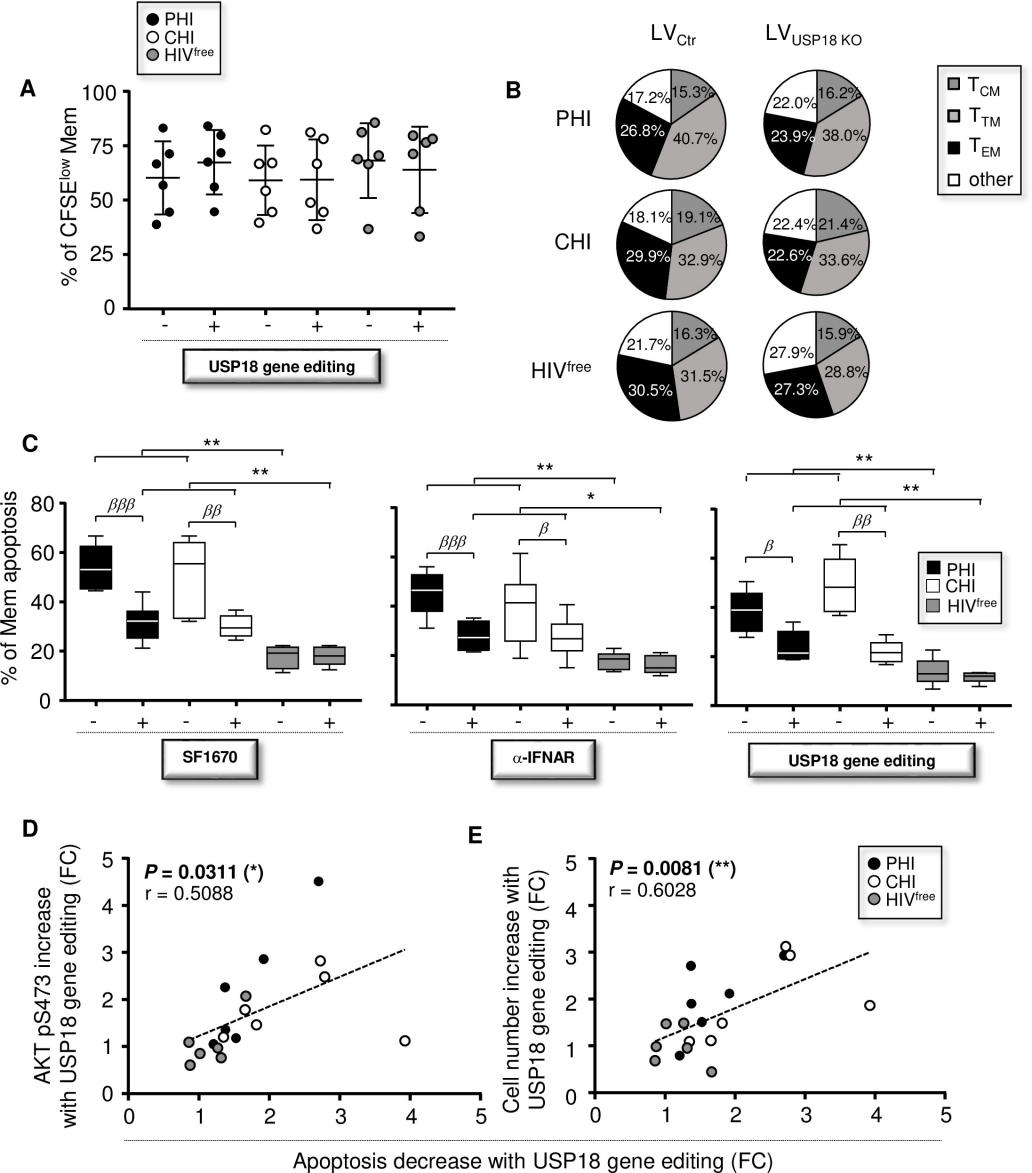
Improvements of Mem maintenance in PHI and CHI with USP18 gene editing are associated with lower cell death. (**A**) Levels of proliferation determined at day 7 of culture by the percentages of viable CFSE^low^ Mem with or without CRISPR/Cas9 mediated USP18 gene editing (n = 6). (**B**) Distribution of Mem subsets at day 7 of culture with or without CRISPR/Cas9 mediated USP18 gene editing (n = 6). (**C**) Percentages of apoptotic Mem on day 7 with or without SF1670 (left), α-IFNAR or its respective isotype control (middle), or CRISPR/Cas9 mediated USP18 gene editing (right) (n = 6). Levels of apoptosis were determined using Annexin-V staining. (**D**) Correlation between the reductions of Mem apoptosis and the increases of AKT pS473 levels after USP18 gene editing in Mem at day 7 of culture (FC, fold change; n = 18). (**E**) Correlation between the reductions of Mem apoptosis and the increases of cell counts after USP18 gene editing treatment at day 7 (FC, fold change; n = 18). The error bars indicate standard deviations from the means. *β*, symbol used for paired *t* test (comparison between treated Mem and control). *, symbol used for Mann-Whitney test (comparison between study groups).

We next aim to identify the main cause of reduced Mem counts in HIV-1-infected subjects at day 7 of culture. To investigate whether reduced numbers of Mem may be explained by lower proliferation rates, we stained purified Mem with carboxyfluorescein succinimidyl ester (CFSE) at day 0. Our data showed comparable percentages of proliferating CFSE^low^ Mem in cultures from PHI, CHI and HIV^free^ subjects (Fig 6A). Similarly, reduced numbers of Mem in cultures from HIV-1-infected subjects at day 7 could not be explained by different cell distribution pattern when compared to HIV^free^ donors. Indeed, we found similar cell distribution among Mem for all study groups as determined by the percentages of T_CM_, T_TM_ and shorted-lived T_EM_ cells [39] (Fig 6B). Although the targeted USP18 editing in HIV-1-infected subjects led to significant increases of Mem at day 7 of culture, it did not impact their levels of proliferation or cell distribution patterns (Fig 6A and 6B). Put together, these results demonstrated that proliferation and cell differentiation did not play a significant role in long-lasting Mem maintenance.

In contrast, we found higher levels of apoptosis in Mem from PHI and CHI subjects compared to HIV^free^ donors at day 7 of culture (38.6 ± 8.4 [PHI], 49.2 ± 11.2 [CHI] and 13.8 ± 5.5 [HIV^free^]) (Fig 6C). Treatments with SF1670, α-IFNAR, and LV_USP18 KO_ also led to significant reduction of Mem apoptosis in PHI and CHI subjects, although percentages of apoptotic Mem in culture from uninfected controls were systematically lower. Finally, we found a positive correlation between the reductions of Mem apoptosis and the increases of AKT pS473 levels after USP18 gene editing in Mem at day 7 of culture (*P* = 0.0311, r = 0.5088; n = 18) (Fig 6D). The reduction of Mem apoptosis following USP18 gene editing also correlated with the increases of cell counts (*P* = 0.0081, r = 0.6028; n = 18) (Fig 6E).

In summary, our data indicate that Mem apoptosis regulates the cell numbers in our long-term culture assay rather than proliferation or cell differentiation.

### Interfering with USP18 expression in primary-infected subjects also rescues HIV-1-specific cells from apoptosis and involves increased AKT pS473 levels

**Fig 7:**
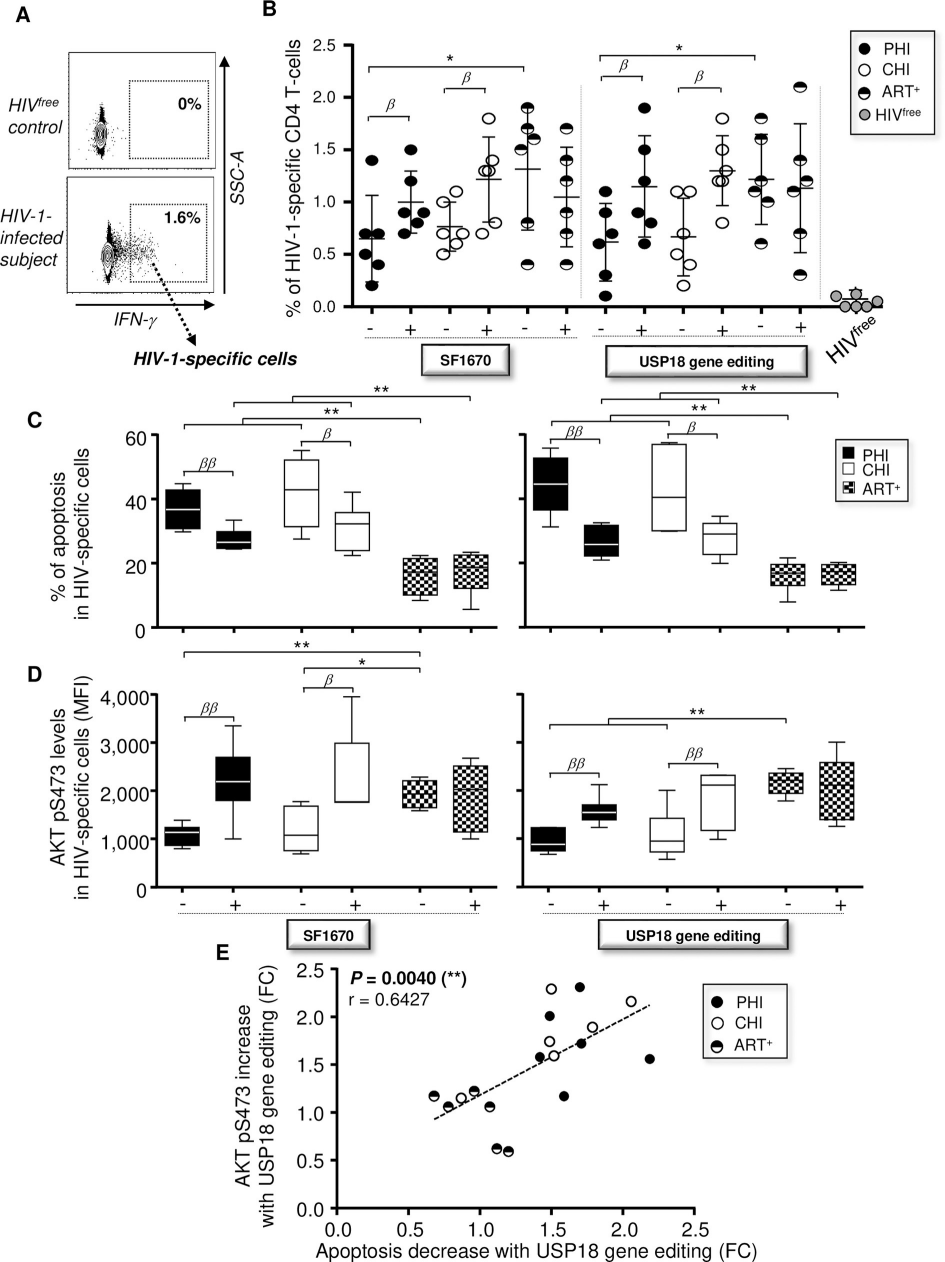
Interfering with USP18 reduces apoptosis of HIV-1-specific CD4 T-cells in an AKT-dependent manner. (**A**) Gating strategy for HIV-1-specific CD4 T-cells following Gag stimulation for 18 hours. HIV-1-specific clones were determined by IFN-γ expression. (**B**) Percentages of HIV-1-specific CD4 T-cells (on total CD4) in CHI, PHI, ART^+^ and HIV^free^ subjects after Gag stimulation in the presence or absence of SF1670. Percentages of HIV-1-specific cells were also determined in culture after 18 hours of Gag stimulation when CD4 T-cells have been pre-transduced or not for 48 hours with LV_USP18 KO_. HIV^free^ donors were included as negative control for HIV-1 stimulation (n = 6). (**C,D**) Levels of apoptosis assessed by Annexin-V staining (**C**) and AKT pS473 expression (**D**) in HIV-1-specific CD4 T-cells at 18 hours post-stimulation in the presence of absence of SF1670 (left) or CRISPR/Cas9 mediated USP18 gene editing (right) (n = 6). (**E**) Correlation between the reductions of apoptosis and increases of AKT pS473 levels in HIV-1-specific CD4 T-cells after USP18 gene editing (FC, fold change; n = 18). The error bars indicate standard deviations from the means. *β*, symbol used for paired *t* test (comparison between treated Mem and control). *, symbol used for Mann-Whitney test (comparison between study groups).

Since evidence show that HIV-1-specific CD4 T-cells display enhanced apoptotic potential that the other Ag-experienced cells [42], we decided to assess if interfering with PTEN or USP18 could also reduce the apoptosis of those cells. First, PBMC from PHI, CHI, and ART^+^ subjects were stimulated with HIV-1 Gag p55 antigens and anti-CD28 Abs for 18 hours in the presence or absence of SF1670 inhibitor. We added the antiviral AZT in cultures from ART^+^ subjects to maintain medical pressure and prevent *de novo* infections and viral replication. The efficacy of AZT treatment was confirmed by the absence of detectable p24 levels in supernatants from ART^+^’s cultures. We also inhibited USP18 expression in Mem from all groups of HIV-1-infected subjects before assessing the Gag-specific stimulation. Briefly, we transduced purified CD4 T-cells with LV_USP18 KO_ or LV_Ctr_ for 4 hours, washed the cells twice, and cultured them for 48 hours with their autologous CD4-depleted PBMC (ratio CD4/PBMC = ¼). Finally, transduced cells were stimulated for an additional 18 hours with Gag antigens and anti-CD28 Abs. At 18 hours post-stimulation with Gag, we collected cells and assessed the expression of USP18, IFN-γ and AKT pS473 as well as the percentages of apoptotic cells by Annexin-V staining within the HIV-1-specific CD4 T-cells. HIV-1-specific CD4 T-cells from infected subjects were determined by their positive staining for IFN-γ following Gag stimulation (Fig 7A). Of note, we included HIV^free^ donors as negative controls for HIV-1-specific stimulations. Uninfected controls were used for setting gating regions and discerning negative from positive cells (HIV^free^: 0.08 ± 0.12% of IFN-γ^+^CD4 T-cells at 18 hours Gag post-stimulation; Fig 7B). Our data showed that PHI displayed lower proportion of HIV-1-specific CD4 T-cells when compared to ART-suppressed subjects after Gag stimulation (*P* = 0.0476, n = 6) (Fig 7B). Although it did not reach significance, we also found a trend to reduced proportion of HIV-1-specific cells in CHI when compared to ART^+^ subjects (0.77 ± 0.23% and 1.32 ± 0.56%, respectively). We confirmed increased USP18 expression in HIV-1-specific CD4 T-cells from PHI and CHI subjects when compared to those from ART^+^ (S13 Fig; cells with no transduction or transduced with LV_Ctr_). We also confirmed approximately 81.2% and 83% inhibitions of USP18 expression in HIV-1-specific CD4 T-cells from PHI and CHI subjects when their purified CD4 T-cells were pre-transduced with LV_USP18 KO_ (S13 Fig). In this context, our results showed that treatment with SF1670 and USP18 gene editing with PHI and CHI subjects led to significant increases of proportion of HIV-1-specific cells (Fig 7B).

Our data further showed increased percentages of apoptotic Annexin-V^+^ HIV-1-specific CD4 T-cells in PHI and CHI subjects when compared to patients under ART (36.8 ± 6.1, 42 ± 10.9 and 16.2 ± 5.9, respectively; *P* = 0.0022) (Fig 7C). As expected, interfering with PTEN or with USP18 expression in viremic subjects led to significant reduction of apoptosis in Gag-specific cells, although levels of apoptosis in PHI and CHI subjects were still higher compared to those of ART^+^ subjects (Fig 7C). We also found reduced expression levels of AKT pS473 in HIV-1-specific CD4 T-cells from viremic subjects compared to ART^+^ patients (*P* = 0.0022 and *P* = 0.0173, respectively; n = 6) (Fig 7D). SF1670 treatment or USP18 gene editing led to increases of AKT activation in PHI and CHI subjects (Fig 7D). Finally, we found a significant correlation between the reductions of apoptosis and increases of AKT pS473 levels in HIV-1-specific CD4 T-cells with USP18 gene editing (*P* = 0.0040, r = 0.6427; n = 18) (Fig 7E).

Overall, our results show that interfering with PTEN or USP18 during primary HIV-1 infection not only improves Mem survival, but also protects HIV-1-specific cells as well.

## Discussion

Type I interferons (IFN-I) are central to the innate immune response against viral infections including HIV-1 [10, 12, 43]. In recent years however, the notion that IFN-I have detrimental effects if they are produced for long periods of time, as is the case during persistent viral infections, has become more evident [22, 43, 44]. Similarly to others [16–21], we found elevated IFN-α levels in plasma during the early and later stages of HIV-1 infection, which correlated with viral loads (Fig 1A and S1 Fig). We also found sustained production of IFN-α and viruses in supernatants in PHI and CHI subjects up to 21 days of our *in vitro* cultures (S12 Fig). However, despite indications of an IFN-I signature in HIV-1 infection [22, 32–34], the molecular mechanisms by which sustained IFN-I signaling negatively impacts the immune system, especially Mem, which are a major target during primary infection, are still unclear.

Here, we provide a molecular mechanism related to the elevated IFN-I signaling in Mem during primary HIV-1 infection that is clearly responsible for their defective cell survival (S14 Fig). In this context, our data reveal that IFNAR blockade in Mem during HIV-1 infection normalized the high expression of the down-stream interferon-induced gene, USP18, in the range of those from uninfected controls (Fig 1D–1F). Our data further shows that PTEN expression was reduced following either IFNAR blockade or specific USP18 gene silencing (Fig 2C and 2D). Similarly, it was previously shown that engineered gain of USP18 expression in human lung cancer cell lines stabilized PTEN protein by preventing its ISGylation post-translational modification pathway [45]. Interfering with PTEN activity or specifically with USP18 expression during HIV-1 infection led to significant improvements of Mem survival in an AKT-dependent manner (Fig 3–6). These improvements were illustrated by increased long-lasting cell maintenance, cell response to γ-chain cytokines and resistance to Fas-induced apoptosis (Figs 2–5) [46]. Other observations have shown that IFNAR blockade also inhibited TRAIL-induced apoptosis in CD4 T-cells during *in vitro* infection, and was associated with reduced frequencies of TRAIL^+^ and apoptotic cells in infected subjects [18, 24, 47]. Although a previous report has indicated that prolonged IFN-α treatment could impairs cytokine-induced AKT activation [48], we show the beneficial impact of blocking this signaling in Mem and HIV-1 specific CD4 T-cells and, for what we believe is the first time, we present an explanation on the molecular mechanism involved in this signaling.

Although our findings lead to a straightforward mechanism behind the deleterious effects of sustained IFN-I signaling on the survival of Mem (S14 Fig), there are still other factors to consider if we want to thoroughly map the molecular network involved during this signaling as well as its full impact. (*i*) One such aspect, is the extent at which increased USP18 expression is found. Other populations, such as CD3^+^CD4^neg^, CD3^neg^CD4^neg^, and monocytes, from PHI and CHI also showed increased USP18 expression compared to the uninfected control group (S15 Fig). This places USP18 as a potential key component of the immune system critical function as it could affect a broad spectrum of cell populations during HIV-1 infection. (*ii*) Another factor to consider is the fact that, USP18 is not only an isopeptidase that stabilizes PTEN expression, but also a negative regulator of IFN-I signaling [31, 49]. In the case of this study however, HIV-1-infected subjects displayed elevated IFN-I signaling despite an increase in USP18 expression. It is important to note that the isopeptidase activity of USP18 is independent of its IFN-I regulatory functions [50]. This might explain how high USP18 expression in Mem during HIV-1 infection could stabilize PTEN expression and be concomitantly associated with sustained IFN-I signaling. Of note, the IFN-I regulatory functions of USP18 depend on its capacity to bind IFNAR2 and inhibit JAK/STAT signaling and involves other factors. Through direct interaction with USP18, the insulin receptor substrate-4 was shown to counteract its inhibitory effect on JAK/STAT signaling [51]. Conversely, STAT2 was shown to be a crucial component of the USP18-mediated suppression of IFN-I signaling [52]. Therefore, it would be interesting to investigate how USP18 impacts IFN-I signaling regulation during HIV-1 infection, and whether STAT2 and/or IRS-4 are involved. (*iii*) An additional aspect to consider in the case of sustained IFN-I signaling, aside from Mem survival, is that of their defective function which appears during the early stage of primary HIV-1 infection. In this regard, HIV-1-infected patients display hyper-activated and exhausted CD4 T-cells that are characterized by poor effector functions and high expression of multiple inhibitory receptors such as programmed cell death 1 [53]. Interestingly, recent data collected by Crawford A. *et al* have shown that CD4 T-cell defective function during HIV-1 infection was associated with an elevated IFN-I-induced transcriptional program. Further experiments are however warranted to know whether we could also counteract CD4 T-cell defective function during HIV-1 infection by specifically interfering with USP18 expression. Although we confirmed reduced effector functions illustrated by decreased cell ability to secrete cytokines and polyfunctionality (i.e. ability to secrete multiple cytokines), a 24 hour-long IFNAR blockade did not rescue these immune defects (S16 Fig). However, the critical role of PI3K/AKT signaling pathway in regulating T-cell function is now well established [54]. Therefore, we cannot exclude the possibility that longer IFNAR blockade or specific targeting of USP18, which are acting through AKT-dependent mechanisms, might improve cell functions during persistent HIV-1 infection as well. (*iv*) The elevated IFN-I signaling and USP18 expression in Mem from HIV-1-infected subjects can be explained by their sustained IFN-α and virus production in the environment (S12A and S12B Fig). Therefore, it is not surprising that long-term ART when administrated early during the first months of primary HIV-1 infection was effective in normalizing both IFN-α production and USP18 expression intrinsic to Mem (S5 Fig). Interfering with IFN-I signaling or specifically with USP18 expression may still be a therapeutic approach to consider in some cases of treated patients. Despite effective control of HIV-1 replication with ART, a minority of treated patients called immune non-responders (INR) fails to show increased CD4 T-cell counts to the level observed in uninfected control donors [55, 56]. INR remain at greater risk for health complications and non-AIDS diseases including cardiovascular disease, liver disease, renal disease, and malignancies when compared to immune responders (IRs) in whom the CD4 T-cell counts are properly restored [57, 58]. Although the molecular mechanisms that are responsible for the lack of CD4 T-cell recovery in INR are still unclear, several pieces of observations have indicated that their CD4 T-cell recovery may be adversely affected by the sustained expression of several ISG [59–61]. (*v*) The effect of USP18 modulation on HIV-1 infectivity remains to be fully explored, especially in humanized mice infected with HIV-1 where IFNAR blockade is associated with reduced T-cell activation [32–34]. It is well-established that CD4 T-cell activation is a key factor in facilitating HIV-1 infection and cell depletion [62, 63]. Therefore, we cannot rule out a potential effect of reduced infectivity to contribute to some of the cell survival as the USP18 targeting should be reducing inflammatory/ISG driven activation as well. In the context of our experiments, the relative short time of treatments enables higher AKT activation and cell survival seemingly independently of viral reinfection as maintaining exogenous IFN-α in culture in the presence of fusion inhibitor T20 did not significantly reduce Mem apoptosis compared to untreated Mem (S12C Fig). However, longer treatments might reduce viral infectivity to a significant degree, thus reducing Mem cell death to even greater extents.

As mentioned earlier, although our group has recently provided evidence that the increased production of tryptophan-related catabolite kynurenine during primary HIV-1 infection affected IL-2-induced STAT5 activation in Mem, interfering with this disturbance was not sufficient to restore proper cell survival [6]. Here, we found that blocking IFN-I signaling or directly interfering with USP18 expression also led to significant improvements of Mem survival during primary HIV-1 infection. The molecular mechanisms described in this study did not seem to involve STAT5 activation since we found that IFNAR blockade in Mem from infected subjects had no effect on IL-2-mediated defective STAT5 phosphorylation (S3B and S8C Figs). In contrast, targeting the increased IFN-I signaling, especially the high USP18 expression in Mem from infected subjects, led to significant increase of AKT activation in a PTEN-dependent manner. Although interfering with IFN-I or specifically with USP18 expression during primary HIV-1 infection significantly improved Mem survival in an AKT-dependent manner, it did not reach the levels that were observable in uninfected controls (Figs 2–5). Similarly, although treatments of HIV-1-specific CD4 T-cells from PHI subjects with USP18 gene editing led to reduced apoptosis levels, these levels were higher compared to those from ART^+^ subjects (Fig 7C). These observations indicate that defective Mem survival during primary HIV-1 infection is a complex mechanism, which may involve independent, but synergic molecular disturbances such as sustained IFN-I signaling and high kynurenine production among others. Of note, the catabolism of tryptophan into kynurenine is known to be mediated by the indoleamine 2,3-dioxygenase (IDO), whose protein expression and activity are found increased during HIV-1 infection [64, 65]. Since previous results showed positive correlation between increased ISG expression and IDO levels in HIV-1-infected subjects [66, 67], we cannot exclude the fact that IFN-I blockade may also be effective in counteracting the heightened production of kynurenines during HIV-1 infection. Assessing whether targeting IFN-I and kynurenine-related pathways simultaneously might have a synergistic effect on Mem survival improvement during HIV-1 infection is also warranted.

Although those results are preliminary, USP18 gene editing in cells extracted from one spleen of an HIV-1-infected patient did comfort our observations with PBMC. Indeed, inhibiting USP18 reduced Mem apoptosis even in HIV-1-specific cells (S17 Fig). It remains important to confirm the role of USP18 in tissues with a great number of patients or by using *in vivo* models, such as humanized mice or non-human primate. Taking this into consideration, it is likely that USP18 plays a role in Mem numbers in lymphoid tissue, although its effect might be dwarfed by other mechanisms, such as pyroptosis, more present in those locations compared to peripheral blood [9, 68].

In summary, our data indicates that the interference of sustained IFN-I in human HIV-1-infected subjects, which leads to a better control of USP18 and PTEN, is a valuable tool to consider for Mem recovery and survival via AKT activation. We acknowledge the fact that proposing such therapeutic strategies to fight HIV-1 infection may also bring some concerns, since they could also be detrimental to the patients by protecting their HIV-1-infected cells and sustaining the latent HIV-1 reservoir [69, 70]. Finally, if such treatments have to be considered one day, great consideration should be given to the timing and duration of the IFNAR blockade in patients, especially when taking into account that this blockade accelerated CD4 T-cell depletion in an acute SIV model [71]. However, our data points to USP18 as an important driver for the detrimental phenotypes observed in Mem from HIV-1-infected subjects. Targeting USP18, specifically its isopeptidase activity, could bypass those unintended consequences, as there are fewer pathways impacted when compared to those regulated by IFN-I signaling.

## Materials and methods

### Ethics statement

All infected patients were participants in the Montreal HIV infection study that received approval from the McGill University Health Centre Ethical Review Board (ethic reference number SL-00.069 [blood] and 2019–5170 [spleen]). All subjects provided an informed and written consent for participation.

### Study population

PBMC and plasma were collected from primary infected patients (PHI), untreated ART-naïve and chronically infected subjects (CHI) and patients under ART (ART^+^) who displayed both viral suppression and full CD4 recovery (> 400 CD4/μl blood post-treatments). Each group of HIV-1-infected patients included in the overall study was homogeneously selected and displayed similar clinical data. Clinical information of all infected patients including viral loads and CD4 counts is summarized in S1 Table. We also selected age-matched uninfected control donors as negative control for HIV-1 infection.

### Products

RPMI-1640 media, FBS, antibiotics and PBS were obtained from Wisent Inc. Recombinant IL-2 and IL-7 cytokines as well as the PTEN inhibitor SF1670 were provided from Sigma Aldrich. Anti-Fas CH11 antibody is from MBL International Corporation. We purchased all antibodies and reagents for flow cytometry from BD Biosciences, except for the antibody to CD45RA-ECD, IFNAR2, IRF7 pS477/479 and USP18, which were from Beckman Coulter, Miltenyi Biotec and Santa Cruz Biotechnology respectively (Table S3). 7-aminoactinomycin D (7-AAD) came from ThermoFisher Neutralizing anti-IFNAR antibody (α-IFNAR; clone MMHAR-2) and respective isotype control were obtained from EMD Millipore. Concentrations of SF1670, α-IFNAR and isotype controls used in our study were 3 μM, 5 μg/mL and 5 μg/mL respectively. Concentration for IL-2 and IL-7 were 25 IU/mL and 0.3 ng/mL, respectively. The fusion inhibitor T20 was purchased from Sigma Aldrich.

### ELISA assay

Plasma and culture supernatant levels of IFN-α were measured by ELISA according to the manufacturer’s instructions (high sensitivity human IFN alpha ELISA kit; PBL Assay Science). We also used the sensitive HIV-1 p24 ELISA kit (Abcam) to determine HIV-1 production in cell cultures.

### Purification of Mem

Mem were purified using the untouched memory CD4 isolation kit (EasySep human memory CD4^+^ T-cell Enrichment Kit; StemCell Technologies) allowing for more than 94.6% purification without any cell stimulation and apoptosis.

### Real-time reverse-transcription (RT)-PCR analysis

Total RNA was isolated from purified Mem using an RNeasy kit according to the manufacturer’s instructions (Qiagen). RNA was then reverse transcribed with oligo(dT) primers and SuperScript II reverse transcriptase (Life Technologies). PCR was performed using Taq polymerase (GE Health-care) using set of primers to evaluate ISG expression. The summary of all primers used in this study is presented in S2 Table. All data are presented as relative quantifications with efficiency correction based on the relative expression of target genes versus the gapdh gene as the reference gene. cDNA was amplified using SyBR Green I OPCR master mix (Applied Biosystems), and all data were collected using the Rotor-Gene RG-3000 (Corbet Research) and analysed by the comparative threshold cycle (C*T*) method using the Rotor Gene Q serie software 2.3.1.

### Western blots

Purified Mem from all groups were subjected to SDS-PAGE and Western blot analysis to assess USP18 expression as previously described [4, 72]. Of note, results are expressed as densitometric quantification of specific bands performed using ImageJ software. The levels of expression of USP18 were normalized to β-actin and were later expressed as the ratio of densitometric values of protein of interest divided by densitometric values of actin within the same blot.

### Transfection and siRNA assays

We first purified 5.10^6^ Mem from all tested groups and electroporated them using Nucleofector II technology according the Amaxa Biosystems manufactor’s protocole. Specific USP18 siRNA and Silencer negative control siRNA were obtained from ThermoFisher Scientific. Of note, 5μg of siRNA were transfected or not for each condition for 2 hours without antibiotics. Purified Mem were thereafter washed twice to remove dead necrotic cells, counted and cultured until 48 hours with their autologous CD4-depleted PBMC (at ratio Mem/PBMC = ¼). At 48 hours post-transfection, some cells were kept to measure USP18 and PTEN protein levels by flow cytometry.

### PhosFlow assays

PhosFlow assays were performed to assess the intracellular expression levels of STAT1 pY701, IRF7 pS477/479, STAT5 pY694, and AKT pS473. Briefly, cellular fixation was done using 4% PFA for 10 minutes at 36°C followed by surface staining for 10 minutes at 4°C. Afterwards, the cellular permeabilization was done using 90% ice cold methanol for 30 minutes at 4°C followed by 30 minutes of intracellular staining in PBS+2% FBS at room temperature. Of note, we systematically titrated all antibodies and washed the cells three times at the end of the protocol to ensure that all background fluorescences were at an appropriately low position on the fluorescence scale. The viability marker 7-AAD was used to exclude dead cells from analyses. BD LSRII Fortessa flow cytometer (BD) was used to collect the data which were analyzed using the DIVA software.

### Intracellular staining assays

Staining assays were performed to assess the intracellular expression levels of PTEN and USP18. The cellular permeabilization was done using 0.25% (W/V) saponine in PBS for 30 minutes at room temperature. The following multi-parameter antibody cocktail was used: anti-CD3-BB515, anti-CD4-BV605, anti-CD45RA-APC-Cy7, anti-PTEN-APC and anti-USP18-Alexa Fluor700. Of note, anti-USP18 IgG_1_κ Abs was conjugated to Alexa700 dye using the Zenon mouse IgG_1_ labeling kit (Life Technologies Inc.) according to the manufacturer’ protocol. The viability marker 7-AAD was used to exclude dead cells from analyses. BD LSRII Fortessa flow cytometer (BD) was used to collect the data which were analyzed using the DIVA software. Once again, we titrated all antibodies and washed the cells multiple times during the staining protocol to minimize all background fluorescences.

### Fas-induced apoptosis and cytokine-mediated Mem protection

We first cultured 10^6^ PBMC with either SF1670, IFNAR or its respective isotype control (rat anti-mouse IgG2a Abs) for 48 hours. We also transfected or not Mem with USP18 siRNA or negative control siRNA for 48 hours. Cells were then treated or not with 1.25 μg/mL of anti-Fas CH11 Abs in the presence or absence of cytokines (IL-2 or IL-7) for an additional 24 hours. We determined in Mem for all tested groups an all conditions both the numbers (N) and percentages of constitutive apoptosis (without any treatments), Fas-induced apoptosis and the cytokine-mediated Mem protections when the cells were stimulated with IL-2 or IL-7. Number of Fas-induced apoptotic Mem were determined by the formula: N of apoptotic Mem with CH11 –N of apoptotic Mem without CH11. Similarly to cell numbers, % of Fas-induced apoptosis were determined by the formula: % of apoptosis in Mem with CH11 –% of apoptosis in Mem without CH11. As a reminder, cytokine-mediated Mem protections were calculated in fold change (FC) with the formula: Number of Fas-induced apoptotic Mem without cytokine / Number of Fas-induced apoptotic Mem with cytokine.

### Production of lentiviral vectors and Mem transduction

To produce lentiviral vectors, we used the packaging plasmid psPAX2 and envelope plasmid pMD2G as previously done [4]. As transfer vector, we used either USP18 CRISPR/Cas9 KO plasmid or its respective negative control plasmid (Santa Cruz Biotechnology; sc-402259 and sc-418922, respectively). Briefly, the recombinant virion particles were produced by transient polyethylenimine co-transfection of 10^7^ 293T cells in 175 cm^3^ flasks using 50 μg transfer vector (USP18 CRISPR/Cas9 KO plasmid or control CRISPR/Cas9 plasmid), 200 μg of psPAX2, and 200 μg of pMD2G. The transfection medium was replaced after 24 hr with fresh serum free DMEM medium (Sigma Aldrich). Viral supernatants were collected at 6 days post-transfection, filtered through a 0.45-mm filter and concentrated 150-fold by centrifuging through filtration columns (Centricon Plus-20, molecular weight cutoff 100 kDa; Millipore) at 3000g at 4°C. The concentrated recombinant virus were stored in -80°C for further usage. Viral titers (ng/mL) were assessed by using HIV-1 p24 ELISA. Of note, we used 100 ng of lentiviral vectors (LV) per 1.10^6^ purified Mem for 4 hours, washed the cells twice and cultured them until 48 hours with their autologous CD4-depleted PBMC (ratio Mem/PBMC = ¼) to achieve significant USP18 inhibition.

### Long-lasting Mem maintenance assays

2.10^4^ purified Mem were first activated with 0.5 μg/mL anti-CD3 and 1 μg/mL anti-CD28 Abs in the presence or absence of ST1670, anti-IFNAR or its respective isotype control for 2 hours. Mem were then washed twice, counted and cultured with 8.10^4^ autologous CD4-depleted PBMC. Cultured cells were re-stimulated with anti-CD3 and anti-CD28 Abs with or without the specific inhibitors at days 7, 14 and 21. To interfere with USP18 expression during the long-term culture, we also purified Mem from all groups, transduced them with LV_USP18 KO_ or LV_Ctr_ for 4 hours. At 4 hours post-transduction, we counted the cells and cultured 2.10^4^ of them with 8.10^4^ autologous CD4-depleted PBMC. Cultured cells were re-stimulated with anti-CD3 and anti-CD28 Abs at days 7, 14 and 21. Total numbers of viable Mem were counted, and the half-lives of these cells were estimated for each study groups at days 7, 14, 21 and 28 of culture. We also determined in gated Mem the levels of apoptosis using Annexin-V staining and the expressions of USP18 and AKT pS473 by flow cytometry. We also assessed by ELISA the levels of both IFN-α and virus productions in supernatants during the time course of culture. Finally, we also determined at day 7 of culture the levels of cell proliferation, differentiation and apoptosis in Mem for all donors as previously done [4].

### HIV-1-specific stimulation

PBMC were specifically stimulated for 18 hours with 5 μg/mL HIV-1 p55 Gag antigens (Austral Biologicals) and 1 μg/mL anti-CD28 Abs in the presence of GolgiPlug and GolgiStop (BD Biosciences). HIV-1-specific stimulations were performed with or without SF1670. We also pre-transduced for 48 hours purified CD4 T-cells from HIV-1-infected subjects before the HIV-1 Gag stimulation. Of note, we added 10 μM AZT (Sigma Aldrich) in cultures from ART^+^ subjects to prevent *de novo* infections (confirmed by HIV-1 p24 ELISA in culture supernatants). Finally, we assessed by flow cytometry the levels of apoptosis using Annexin-V staining and AKT pS473 in responsive IFN-γ^+^ HIV-1-specific CD4 T-cells. Of note, we included HIV^free^ donors as negative controls for HIV-1-specific stimulations. Uninfected controls were used for setting gating regions and discerning positive from negative cells. BD LSRII Fortessa flow cytometer (BD) was used to collect the data which were analyzed using the DIVA software.

### Spleen processing and cell isolation

Spleen tissue was processed within 30 min of surgery (patient info: 47 year old, VL = 3.21 Log copies/ml, CD4 count = 611 cells/μl and CD8 = 1513 cells/μl, 10 years of infection). Blocks were cut into small pieces and forced through a 70μm sterile filter using the plunger of a syringe. Filtrate was kept at 4°C for 2 hours until further processing. Unfiltrated tissue was dissociated enzymatically by digestion with Liberase DL (Roche, Laval, QC, Canada) at 0.1 mg/ml for 1h at 37°C. The digestion material was diluted 3 fold with PBS containing 2% fetal bovine serum (FBS). Mononuclear cells were then isolated from splenocyte filtrate or tissue suspension by centrifugation other ficoll (Wisent, Saint-Jean-Baptiste, QC Canada). Splenocytes were then counted using 0.2% trypan blue to evaluate viability (around 85%), and finally frozen in FBS containing 10% DMSO for further use.

### Statistical analysis

We used the non-parametric Mann-Whitney *U* test that assumes independent samples for all statistical analyses between study groups of subjects (* symbol). On the other hand, statistical analyses between two different *in vitro* conditions were performed using two-sided Student paired *t* test. Spearman’s correlation test was used to identify association among study clinical and immunological variables (β symbol). *P* values of less than 0.05 were considered significant. Of note, several symbols were used depending the statistical analyses. One symbol, 0.05 > *P* > 0.01; two symbols, 0.01 > *P* > 0.001; three symbols, 0.001 > *P* > 0.0001; and four symbols, *P* < 0.0001.

## Supporting information

S1 Table Clinical and immunological data of all selected HIV-1-infected subjects including viral loads and absolute numbers of CD4 counts for 10 PHI, 10 CHI and 10 ART+ subjects. (TIF)

S2 Table Primers used for the real time RT-PCR. (TIF)

S3 Table List of all antibodies used (including information about the vendor, clone IDs and fluorophore). (TIF)

S1 Fig Increased plasma IFN-α in HIV-1-infected subjects correlates with viral load. (**A**) Correlation between viral load (VL; Log_10_) and plasma IFN-α (pg/mL) levels in HIV-1-infected subjects. (n = 19)
(TIF)

S2 Fig Increased levels of IFN-I signaling in CD45RA^+^ CD4 T-cells during HIV-1 infection are not associated with cell loss. (**A**) Gating strategy to define total Mem, T_CM_, T_TM_ and T_EM_ subsets. (**B**) % of STAT1 pY701^+^ (left) or IRF7 pS477/S479^+^ (right) cells on total, CD45RA^+^ and Mem CD4 T-cells in PHI, CHI and HIV^free^ subjects determined by PhosFlow (n = 10). (**C**) Correlations between phospho-protein levels (MFI) and cell percentages in total, CD45RA^+^ and Mem CD4 T-cells (n = 30). The error bars indicate standard deviations from the means. *, symbol used for Mann-Whitney test (comparison between study groups).
(TIF)

S3 Fig Mem from all study groups of subjects displayed similar expression levels for total STAT1 and IRF-7 expression. (**A**) Expression of STAT1 pS727 including representative histograms in Mem from PHI, CHI and HIV^free^ subjects. (**B**) mRNA expression of STAT5 and AKT in unstimulated and cytokine-stimulated Mem. N = 10. The error bars indicate standard deviations from the means. *, symbol used for Mann-Whitney test (comparison between study groups).
(TIF)

S4 Fig Western blot analyses confirmed increased constitutive expression of USP18 in Mem from PHI and CHI subjects when compared to HIV^free^ controls. (**A**) % of *ex vivo* USP18^+^ Mem in PHI, CHI and HIV^free^ (n = 10). (**B, C**) USP18 expression determined in *ex vivo* Mem by western blot (n = 4). (**B**) Representative blots for USP18 and β-actin (sampling n2). (**C**) Densitometric quantification of USP18 expression with four sampling (PHI, CHI and HIV^free^ control). Results shown represent the USP18 relative expression after β-actin normalization in each sampling. *, symbol used for Mann-Whitney test (comparison between study groups).
(TIF)

S5 Fig ART when administrated early and after years of treatment normalizes IFN-α production and IFN-I signaling intrinsic to Mem. (**A**) Plasma concentration of IFN-α in ART^+^ and HIV^free^ subjects determined by ELISA (pg/mL). (**B**) Expression levels of USP18 on *ex vivo* Mem from ART^+^ and HIV^free^ subjects in MFI (***i***) or percentages of USP18^+^ Mem (***ii***). (**C**) Expression levels of PTEN on *ex vivo* Mem from ART^+^ and HIV^free^ subjects in MFI (***i***) or percentages of USP18^+^ Mem (***ii***). (**D**) *In vitro* AKT pS473 expression levels in Mem in the presence or absence of cytokine stimulations in MFI (***i***) or percentages of USP18^+^ Mem (***ii***). (**A-D**) (n = 10). The error bars indicate standard deviations from the means. *, symbol used for Mann-Whitney test (comparison between study groups).
(TIF)

S6 Fig *Ex vivo* Mem from PHI, CHI and HIV^free^ subjects display similar IFNAR expression and subset distribution. (**A,B**) *Ex vivo* IFNAR1 and IFNAR2 surface expression in Mem determined as percentages of positive cells (**A**) and mean fluorescence intensities or MFI (**B**). (**C**) *Ex vivo* distribution of Mem subsets. Representative pie charts for each study group of subjects are shown above. (**A-C**) (n = 10). The error bars indicate standard deviations from the means. *, symbol used for Mann-Whitney test (comparison between study groups).
(TIF)

S7 Fig Specific USP18 gene silencing led to significant inhibition of its protein expression in Mem from HIV-1-infected subjects. (**A**) % of *ex vivo* PTEN^+^ Mem in PHI, CHI and HIV^free^. (**B**) USP18 Expression levels in Mem following 48 hours of specific USP18 siRNA transfection in PHI, CHI and HIV^free^ subjects (MFI). Representative histograms including isotype control and transfected Mem for one PHI are also shown on the right side (MFI and % of positive cells). (**C**) PTEN expression in Mem that have been electroporated alone or transfected with scrambled siRNA. (**A-C**) (n = 10). The error bars indicate standard deviations from the means. *β*, symbol used for paired *t* test (comparison between treated Mem and control). *, symbol used for Mann-Whitney test (comparison between study groups).
(TIF)

S8 Fig Interfering with IFN-I signaling in Mem does not improve IL-2-mediated STAT5 activation. (**A,B**) Expression levels of STAT5 pY694 and AKT pS473 on Mem following 15 minutes of IL-2 or IL-7 stimulation determined as (**A**) percentages of positive cells and (**B**) mean fluorescence intensities or MFI. (**C**) PBMC were first incubated overnight with α-IFNAR or respective isotype control, and then stimulated with IL-2 for another 15 minutes before assessing STAT5 activation levels by PhosFlow (MFI). (**A-C**) (n = 10). The error bars indicate standard deviations from the means. *, symbol used for Mann-Whitney test (comparison between study groups).
(TIF)

S9 Fig Interfering with USP18 in Mem from PHI and CHI improves cell resistance to apoptosis as determined by the percentages of apoptosis. (**A**) Percentage of Fas-induced apoptosis in Mem in the presence or absence of IL-2 or IL-7 stimulation. Fas-induced apoptosis was calculated according the formula: % of apoptosis in Mem with CH11 –% of apoptosis in Mem without CH11 (n = 10). (**B**) Number of Fas-induced apoptotic Mem in the presence or absence of IL-2 or IL-7 stimulation in Mem that have been pre-treated for 48h with SF1670 (*i*), α-IFNAR or its respective isotype control (*ii*), or pre-transfected or not for 48 hours with USP18 siRNA (*iii*). Number of Fas-induced apoptototic Mem was calculated according the formula: N of apoptotic Mem with CH11 –Number of apoptotic Mem without CH11 (n = 10). The error bars indicate standard deviations from the means. *β*, symbol used for paired *t* test (comparison between treated Mem and control). *, symbol used for Mann-Whitney test (comparison between study groups).
(TIF)

S10 Fig USP18 siRNA transfection in Mem does not impact their CD95 expression levels. (**A,B**) Expression levels of CD95 in Mem that have been pre-transfected 48 hours with specific USP18 siRNA or scramble control. Results are expressed as (**A**) mean fluorescence intensities and (**B**) percentages of positive cells. (**A,B**) (n = 10). The error bars indicate standard deviations from the means.
(TIF)

S11 Fig CRISPR/Cas9 mediated USP18 gene editing in Mem results in significant and sustained inhibition of USP18 expression. (**A**) USP18 expression in Mem that have been transduced or not for 48 hours with lentiviral CRISPR/Cas9 vectors mediating USP18 gene editing (lentiviral vectors for USP18 knock-out or LV_USP18 KO_) or control lentiviral vectors (LV_Ctr_) in PHI, CHI and HIV^free^ subjects (MFI; n = 10). Representative histograms including isotype control are also shown on the right side for one PHI subjects. (**B**) USP18 expression in Mem from PHI, CHI and HIV^free^ subjects following Mem TcR activation every 7 days for 28 days in the presence or absence of CRISPR/Cas9 mediated USP18 gene editing (n = 6). The error bars indicate standard deviations from the means. *β*, symbol used for paired *t* test (comparison between treated Mem and control). *, symbol used for Mann-Whitney test (comparison between study groups).
(TIF)

S12 Fig Specifically interfering with USP18 expression in Mem does not impact IFN-α secretion or virus production during long-lasting *in vitro* assay. (**A**) IFN-α and (**B**) p24 levels in culture medium from PHI and CHI subjects at day 7, 14 and 21 days of culture when Mem have been transduced or not at day 0 with LV_USP18 KO_. Results are expressed in pg/mL (n = 6). (**C**) Apoptosis levels in Mem from PHI and CHI at day 7 of culture when treated or not with two antiretrovirals (ARV) (n = 6). In this context, we used 10 μM AZT and one fusion inhibitor to prevent any *de novo* infection (100 nM T20). We also added or not at day 0 of cultures 150 IU/ml IFN-α to sustain IFN-I signaling in the absence of virus. The levels of HIV-1 p24 in pg/ml assessed at day 7 in supernatants are also indicated in bold for all conditions (Und., undetectable levels). The error bars indicate standard deviations from the means. *β*, symbol used for paired *t* test (comparison with no treatment or NT).
(TIF)

S13 Fig Significant inhibition of USP18 expression levels in HIV-1-specific CD4 T-cells with CRISPR/Cas9 mediated USP18 gene editing. USP18 expression levels (MFI) in IFNγ^+^ HIV-1-specific CD4 T-cells following 18 hours of Gag stimulation when cells have been pre-transduced or not with LV_USP18 KO_. Representative histograms including isotype control are also shown on the right side for one PHI. (n = 10). The error bars indicate standard deviations from the means. *β*, symbol used for paired *t* test (comparison between treated Mem and control). *, symbol used for Mann-Whitney test (comparison between study groups).
(TIF)

S14 Fig Interplay between sustained IFN-I signaling, USP18 expression and reduced AKT activation in memory CD4 T-cells during HIV-1 infection. (TIF)

S15 Fig USP18 expression is increased in other cell populations during HIV-1 infection. Constitutive expression of UPS18 is higher in CD3^+^CD4^neg^, CD3^neg^CD4^neg^ and monocytes from PHI and CHI when compared to HIV^free^ subjects as determined with MFI values (left) and percentages of positive cells (right) (n = 10). Representative histograms including isotype control are also shown below. The error bars indicate standard deviations from the means. *, symbol used for Mann-Whitney test (comparison between study groups).
(TIF)

S16 Fig Functional defects observed in viremic HIV-1-infected subjects are not rescued by IFNAR blockade. (**A**) Results are shown as the increases in FC (Fold change) of TCR-induced secreting Mem in the presence or absence of IFNAR blockade. Secreting cells are defined as producing either IFN-γ, TNF-α, IL-2, or combinations of multiple of them. No statistical differences were observed in the percentage of secreting Mem in the absence of TCR activation for all study groups of subjects. (**B**) Results shown are the increases in FC of TCR-induced IFN-γ producing Mem in the presence or absence of IFNAR blockade. (**C**) Percentages of PD-1 positive cells in Mem in the presence or absence of IFNAR blockade. (**D**) Representative distribution of secreting PD-1 positive Mem in the presence or absence of IFNAR blockade. (**A-D**) N = 10. The error bars indicate standard deviations from the means. *β*, symbol used for paired *t* test (comparison between Mem treated α-IFNAR and IgG2a controls). *, symbol used for Mann-Whitney test (comparison with HIV^free^ controls).
(TIF)

S17 Fig Targeting USP18 expression in spleen CD4 T-cells from an HIV-1-infected subject results in reduced apoptosis. Briefly, spleen cells were collected and transduced for 48 hours with LV_Ctr_ or LV_USP18 KO_. Transduced cells were then activated or not using anti-CD3 and anti-CD28 Abs (TCR stimulation), or p55 Gag and anti-CD28 Abs (HIV stimulation). (**A**) Gating strategy to detect the virus-specific CD4 T-cells at 18 hours of HIV-1 stimulation using IFN-γ expression. (**B**) USP18 expression in gated Mem after 48 hours of cell transduction. Isotype control is also shown in grey. (**C**) Levels of apoptosis on transduced CD4 T-cells after cell activation. Representative histograms show the apoptosis in Mem and IFN-γ^+^ virus-specific cells for TCR and HIV stimulation, respectively.
(TIF)
