# Supplementary figures and images for: USP18 is a significant driver of memory CD4 T-cell reduced viability caused by type I IFN signaling during primary HIV-1 infection
Source: PLoS Pathog. 2019 Oct 28;15(10):e1008060. doi: 10.1371/journal.ppat.1008060 (PMC6837632; doi:10.1371/journal.ppat.1008060)

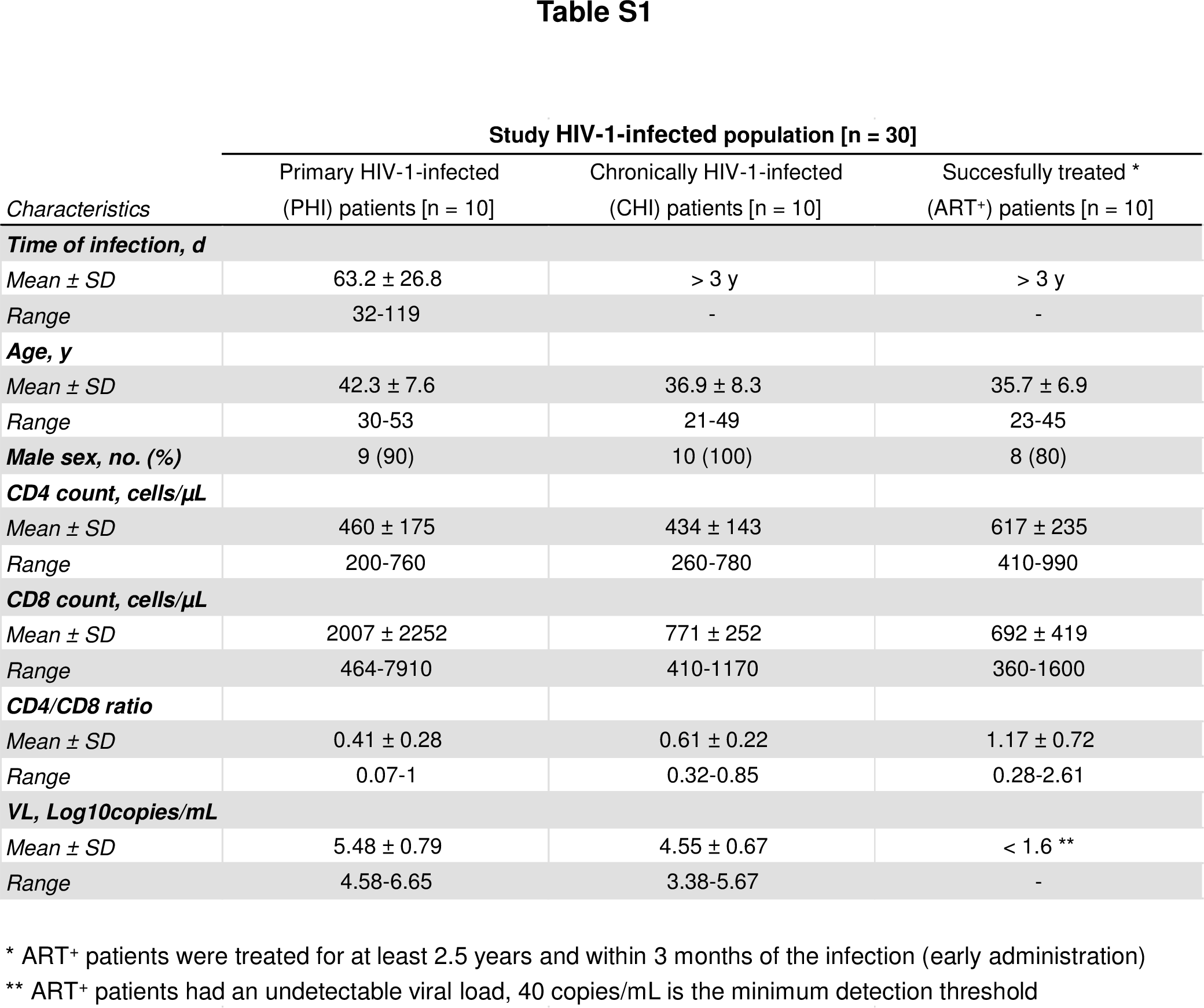

Supplement: S1 Table — (TIF) [file ppat.1008060.s001.tif]

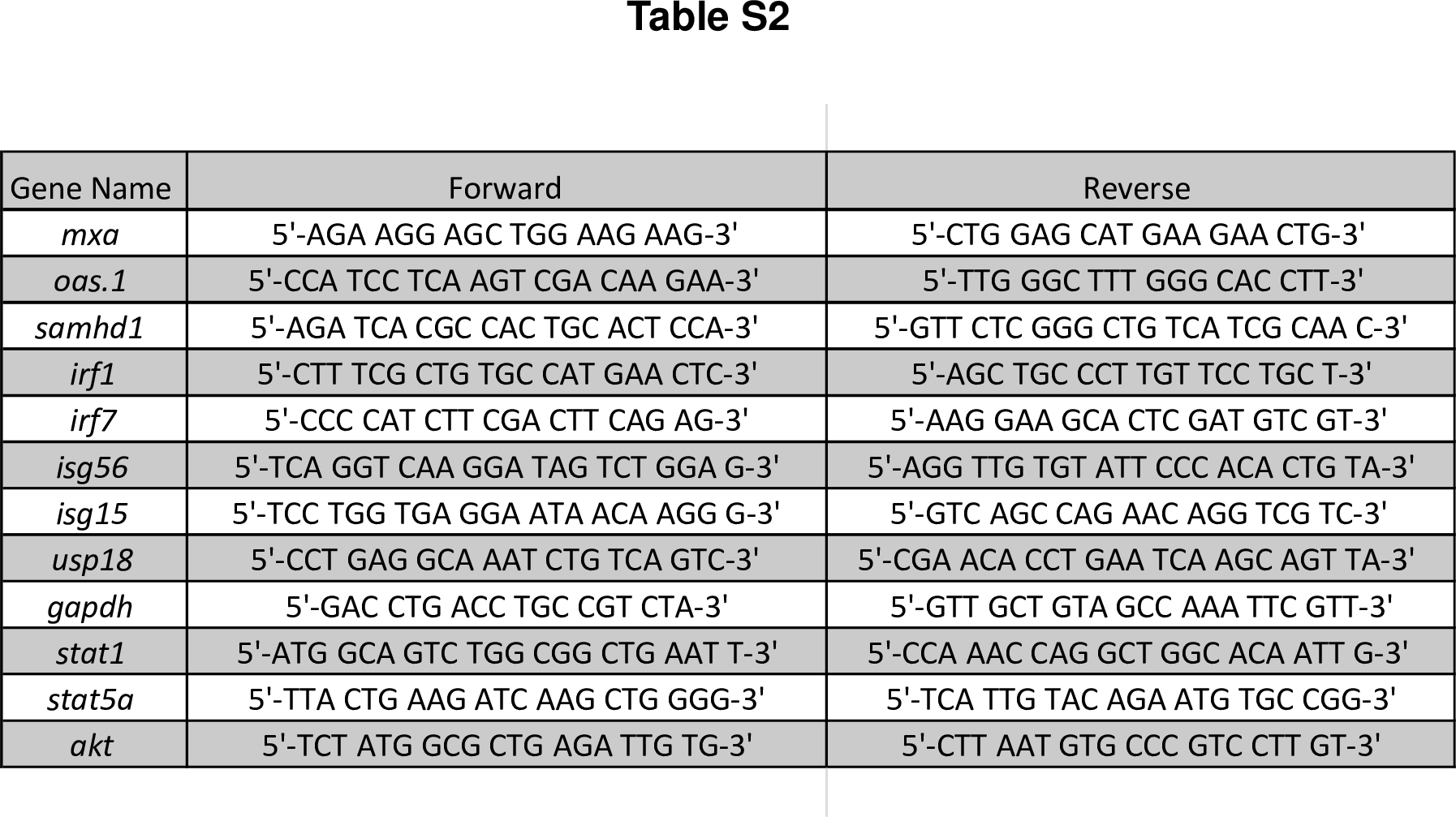

Supplement: S2 Table — (TIF) [file ppat.1008060.s002.tif]

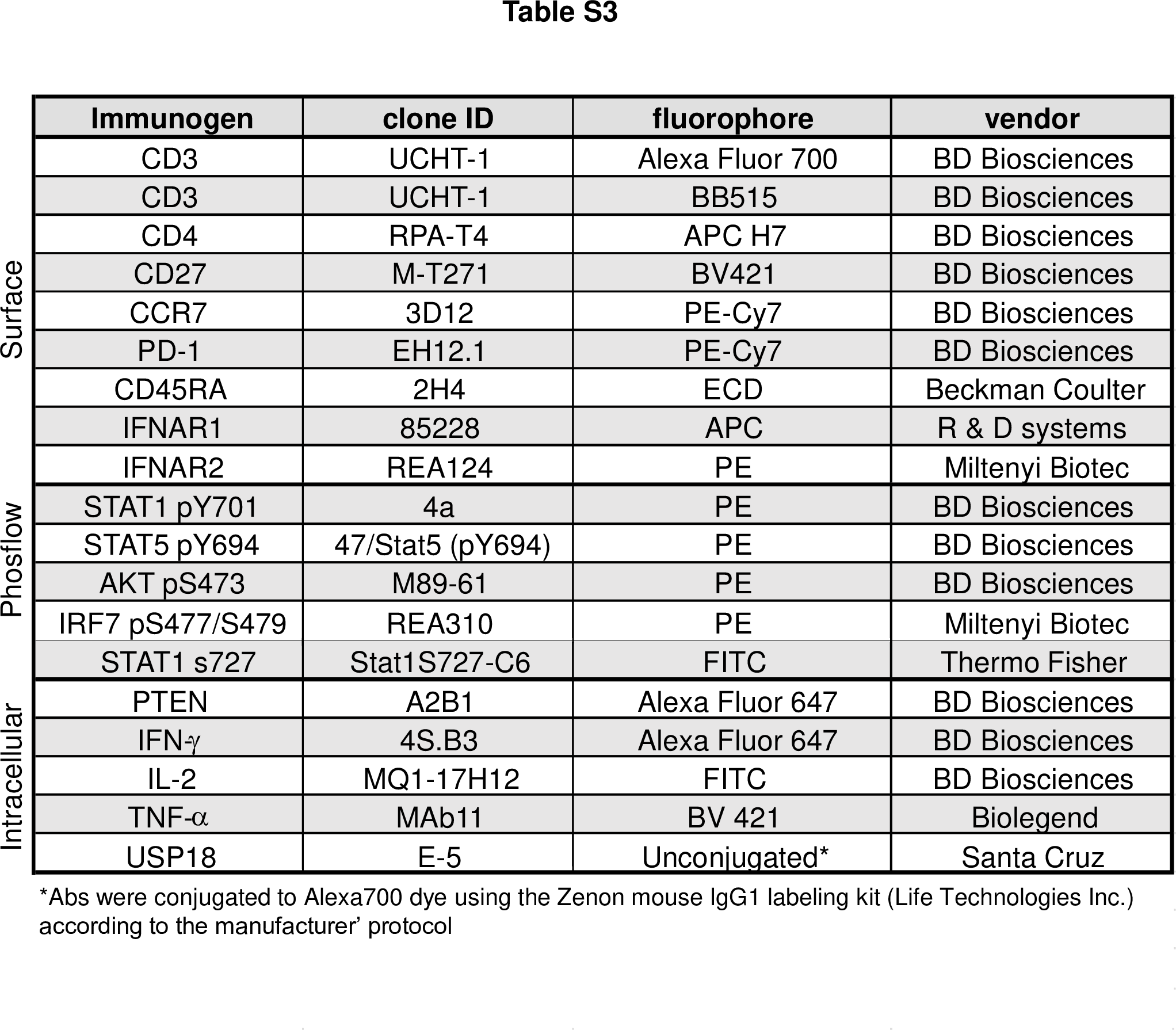

Supplement: S3 Table — (TIF) [file ppat.1008060.s003.tif]

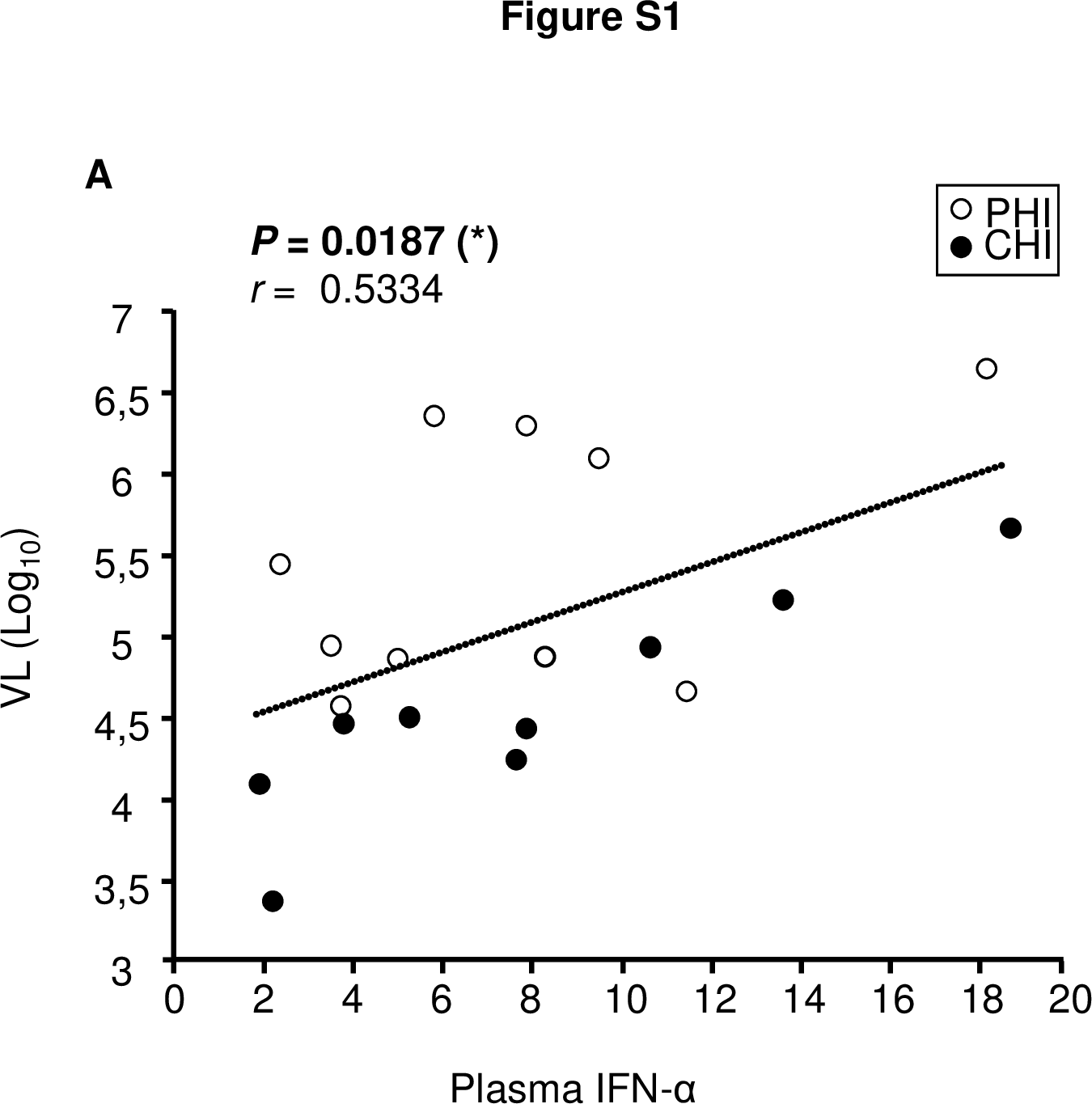

Supplement: S1 Fig — (A) Correlation between viral load (VL; Log10) and plasma IFN-α (pg/mL) levels in HIV-1-infected subjects. (n = 19) (TIF) [file ppat.1008060.s004.tif]

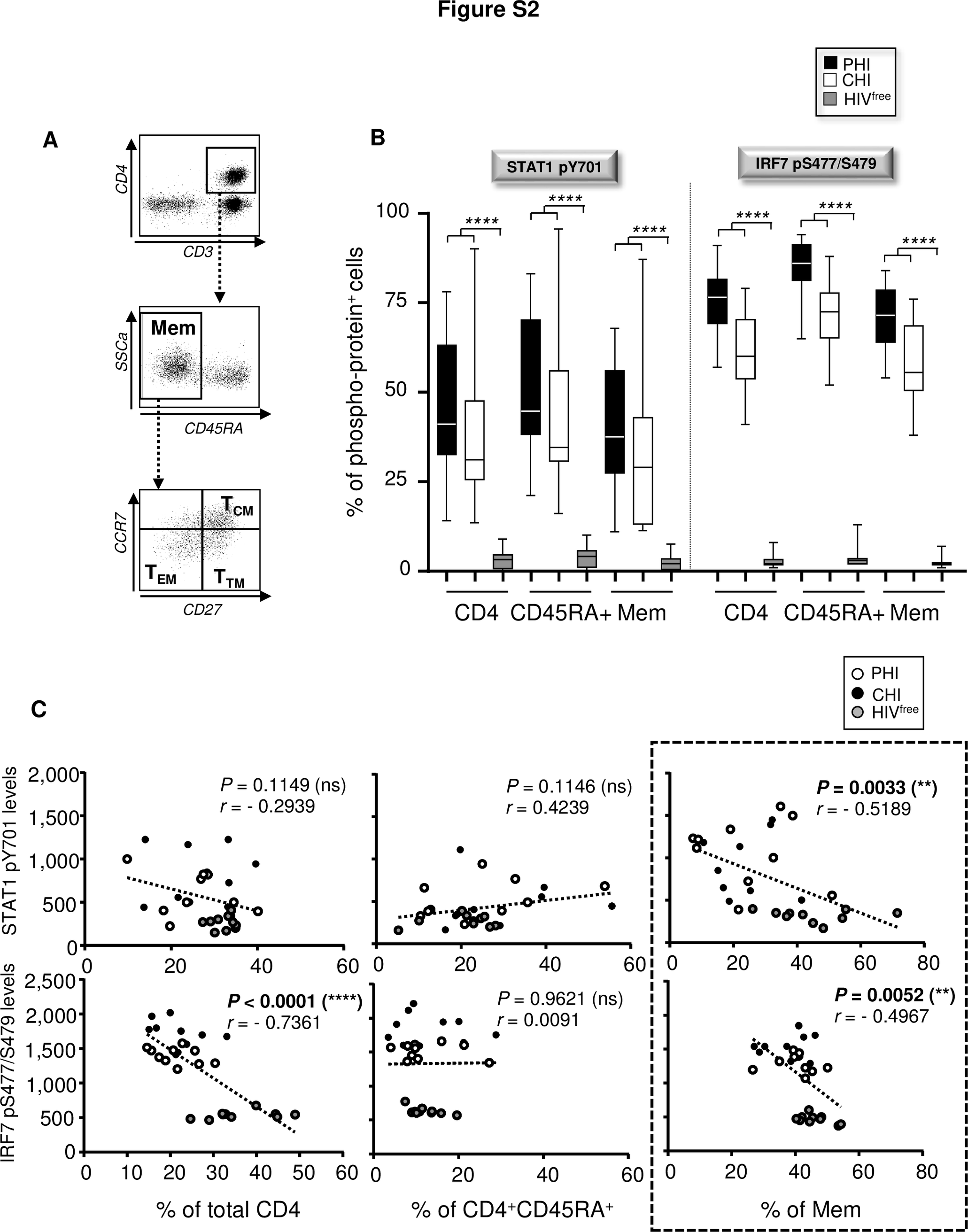

Supplement: S2 Fig — (A) Gating strategy to define total Mem, TCM, TTM and TEM subsets. (B) % of STAT1 pY701+ (left) or IRF7 pS477/S479+ (right) cells on total, CD45RA+ and Mem CD4 T-cells in PHI, CHI and HIVfree subjects determined by PhosFlow (n = 10). (C) Correlations between phospho-protein levels (MFI) and cell percentages in total, CD45RA+ and Mem CD4 T-cells (n = 30). The error bars indicate standard deviations from the means. *, symbol used for Mann-Whitney test (comparison between study groups). (TIF) [file ppat.1008060.s005.tif]

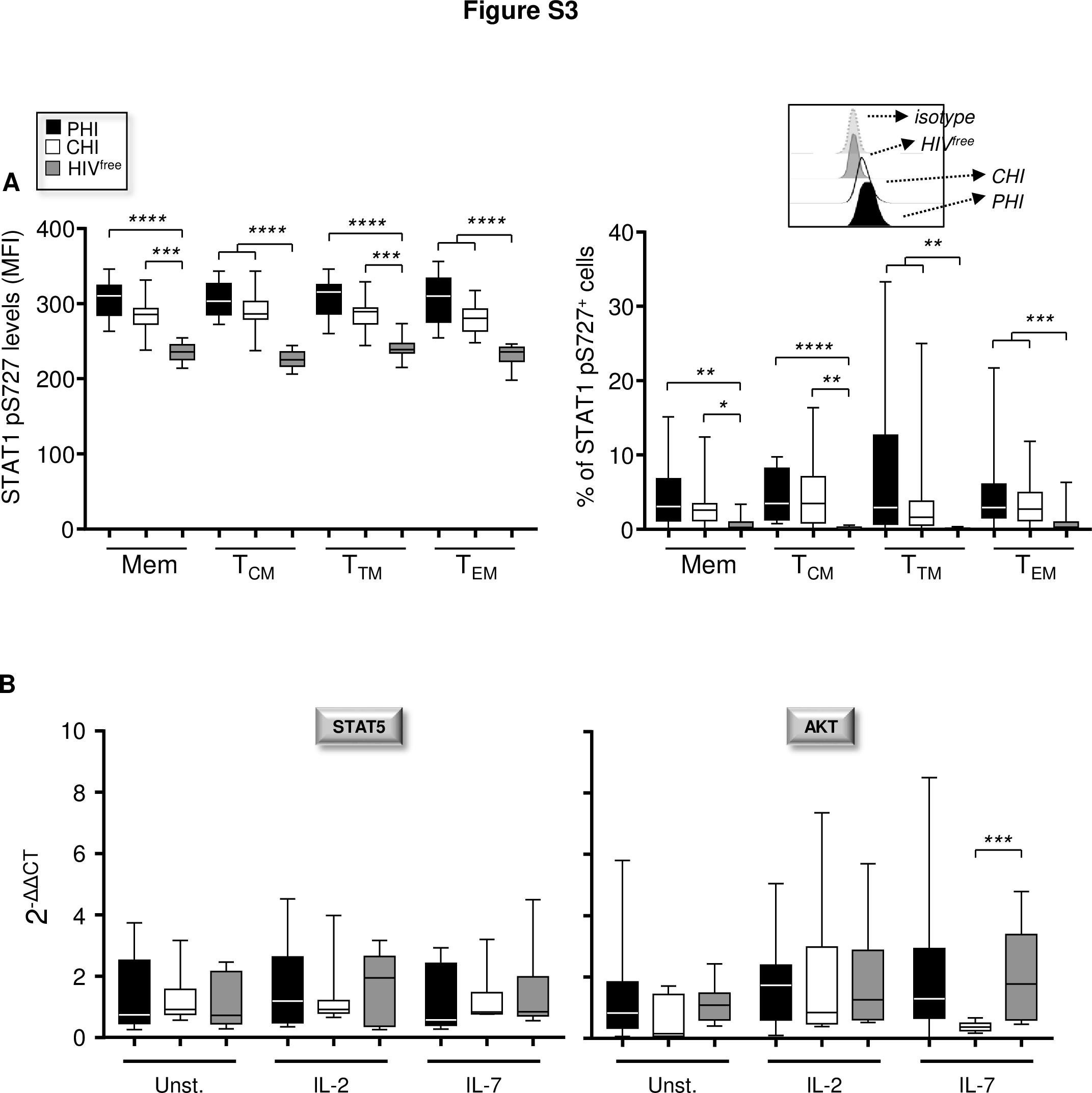

Supplement: S3 Fig — (A) Expression of STAT1 pS727 including representative histograms in Mem from PHI, CHI and HIVfree subjects. (B) mRNA expression of STAT5 and AKT in unstimulated and cytokine-stimulated Mem. N = 10. The error bars indicate standard deviations from the means. *, symbol used for Mann-Whitney test (comparison between study groups). (TIF) [file ppat.1008060.s006.tif]

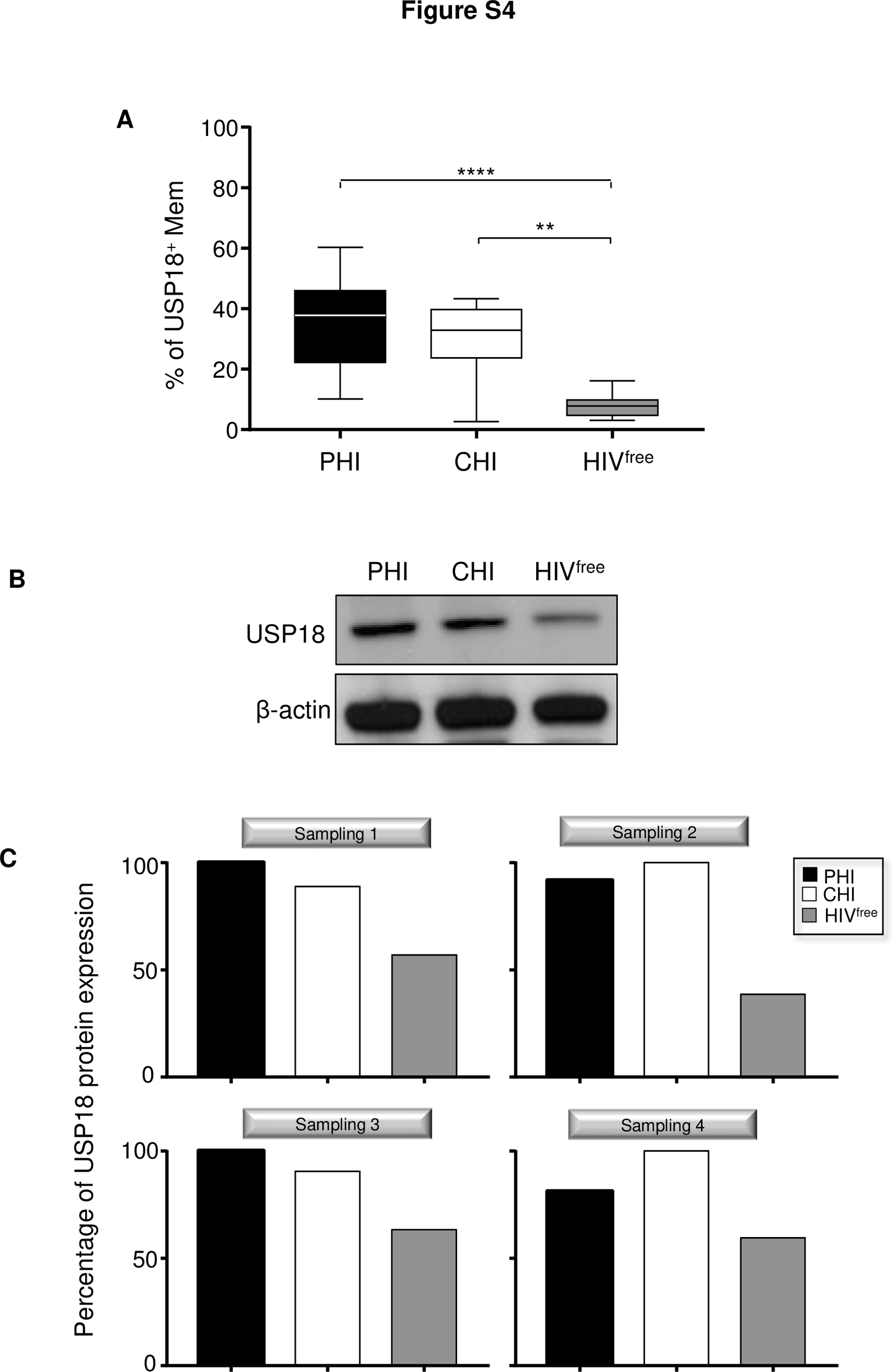

Supplement: S4 Fig — (A) % of ex vivo USP18+ Mem in PHI, CHI and HIVfree (n = 10). (B, C) USP18 expression determined in ex vivo Mem by western blot (n = 4). (B) Representative blots for USP18 and β-actin (sampling n2). (C) Densitometric quantification of USP18 expression with four sampling (PHI, CHI and HIVfree control). Results shown represent the USP18 relative expression after β-actin normalization in each sampling. *, symbol used for Mann-Whitney test (comparison between study groups). (TIF) [file ppat.1008060.s007.tif]

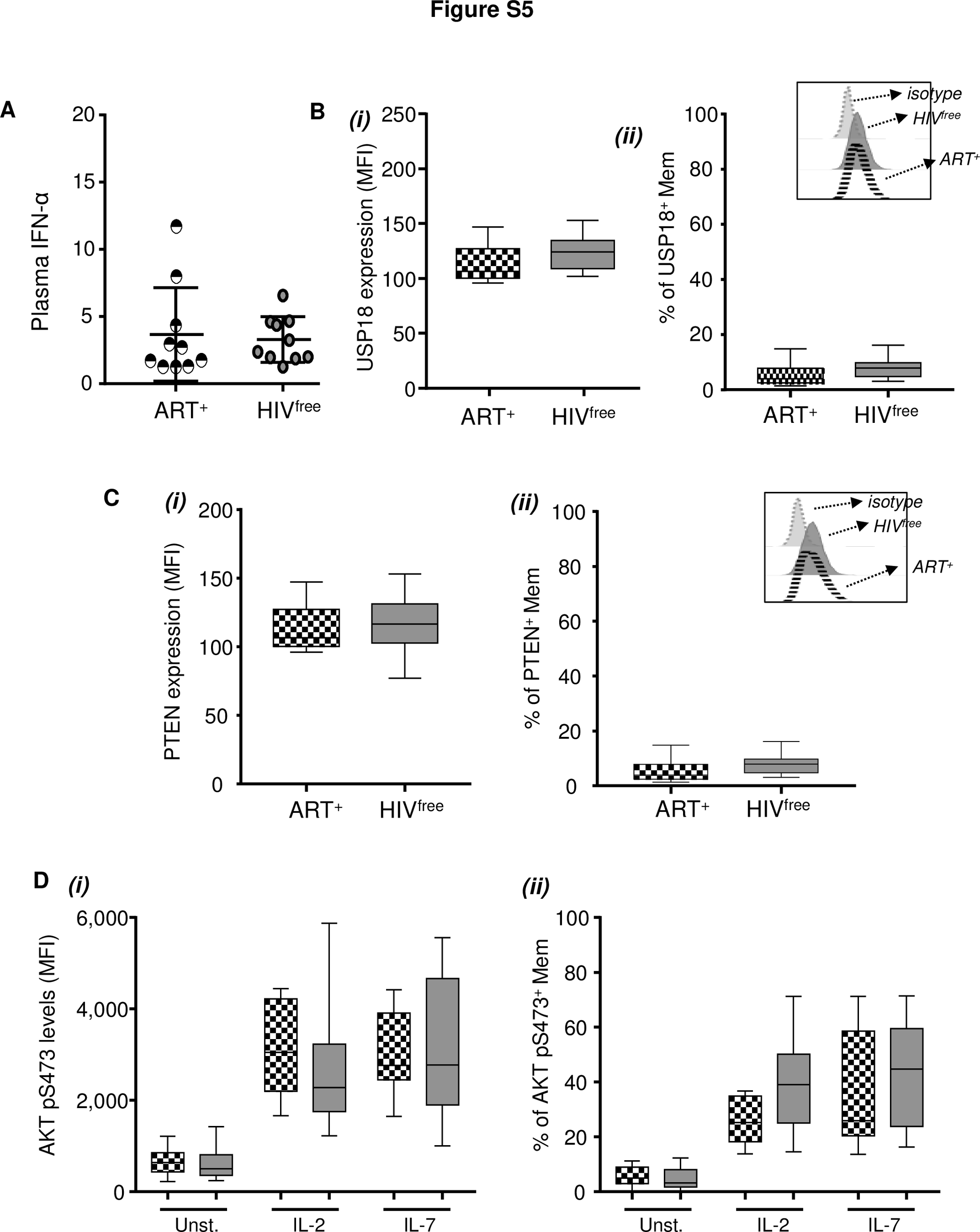

Supplement: S5 Fig — (A) Plasma concentration of IFN-α in ART+ and HIVfree subjects determined by ELISA (pg/mL). (B) Expression levels of USP18 on ex vivo Mem from ART+ and HIVfree subjects in MFI (i) or percentages of USP18+ Mem (ii). (C) Expression levels of PTEN on ex vivo Mem from ART+ and HIVfree subjects in MFI (i) or percentages of USP18+ Mem (ii). (D) In vitro AKT pS473 expression levels in Mem in the presence or absence of cytokine stimulations in MFI (i) or percentages of USP18+ Mem (ii). (A-D) (n = 10). The error bars indicate standard deviations from the means. *, symbol used for Mann-Whitney test (comparison between study groups). (TIF) [file ppat.1008060.s008.tif]

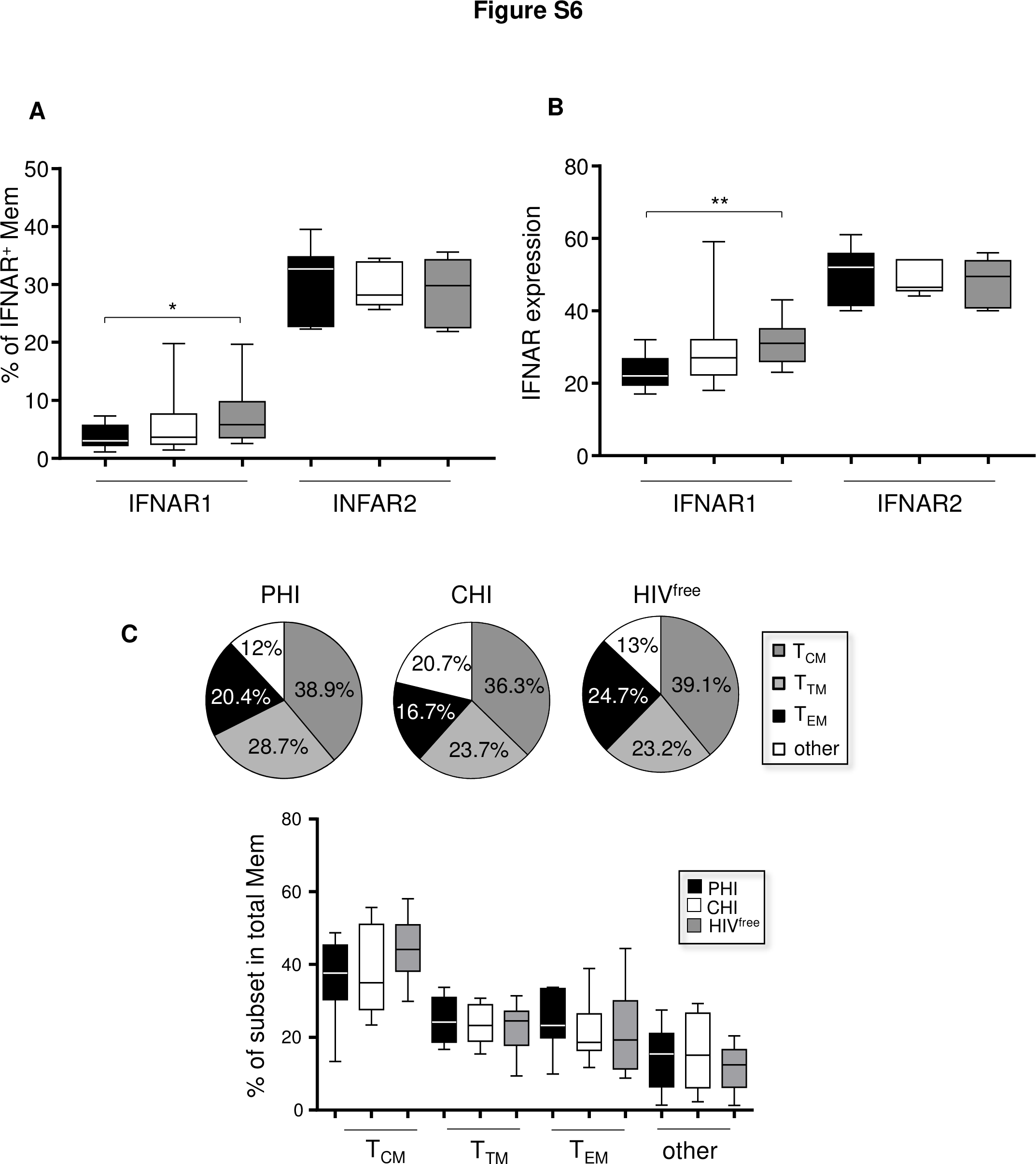

Supplement: S6 Fig — (A,B) Ex vivo IFNAR1 and IFNAR2 surface expression in Mem determined as percentages of positive cells (A) and mean fluorescence intensities or MFI (B). (C) Ex vivo distribution of Mem subsets. Representative pie charts for each study group of subjects are shown above. (A-C) (n = 10). The error bars indicate standard deviations from the means. *, symbol used for Mann-Whitney test (comparison between study groups). (TIF) [file ppat.1008060.s009.tif]

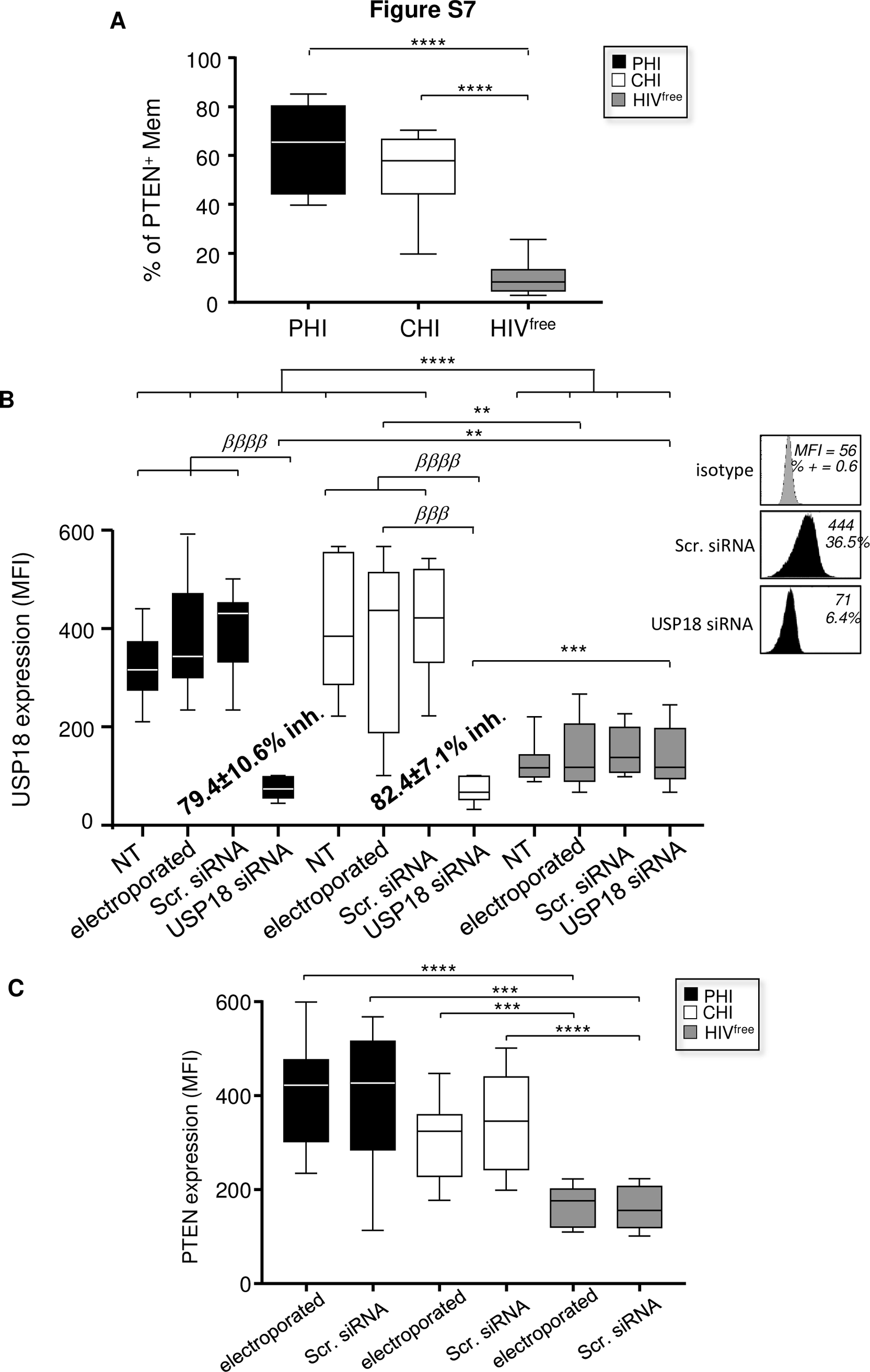

Supplement: S7 Fig — (A) % of ex vivo PTEN+ Mem in PHI, CHI and HIVfree. (B) USP18 Expression levels in Mem following 48 hours of specific USP18 siRNA transfection in PHI, CHI and HIVfree subjects (MFI). Representative histograms including isotype control and transfected Mem for one PHI are also shown on the right side (MFI and % of positive cells). (C) PTEN expression in Mem that have been electroporated alone or transfected with scrambled siRNA. (A-C) (n = 10). The error bars indicate standard deviations from the means. β, symbol used for paired t test (comparison between treated Mem and control). *, symbol used for Mann-Whitney test (comparison between study groups). (TIF) [file ppat.1008060.s010.tif]

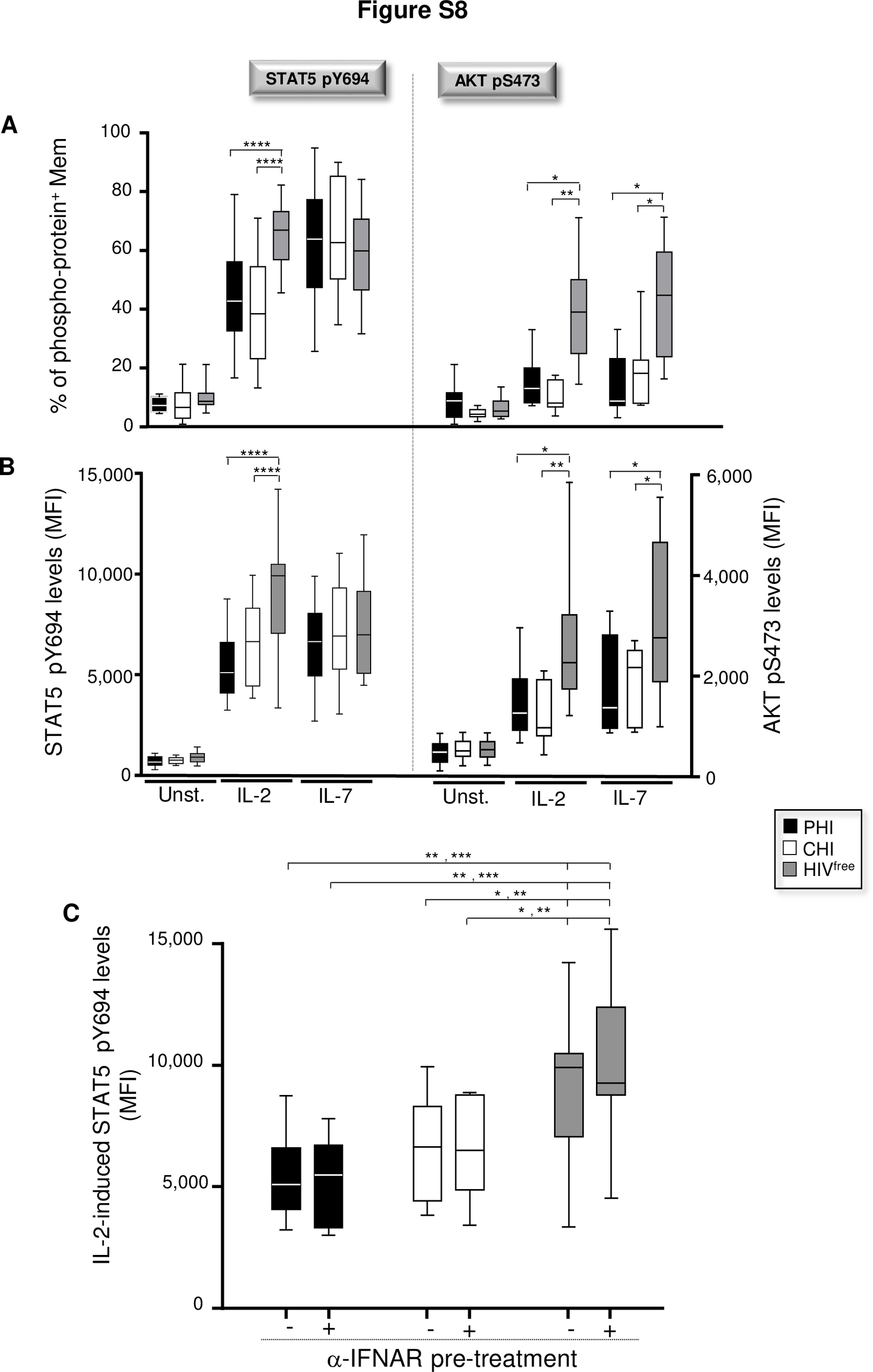

Supplement: S8 Fig — (A,B) Expression levels of STAT5 pY694 and AKT pS473 on Mem following 15 minutes of IL-2 or IL-7 stimulation determined as (A) percentages of positive cells and (B) mean fluorescence intensities or MFI. (C) PBMC were first incubated overnight with α-IFNAR or respective isotype control, and then stimulated with IL-2 for another 15 minutes before assessing STAT5 activation levels by PhosFlow (MFI). (A-C) (n = 10). The error bars indicate standard deviations from the means. *, symbol used for Mann-Whitney test (comparison between study groups). (TIF) [file ppat.1008060.s011.tif]

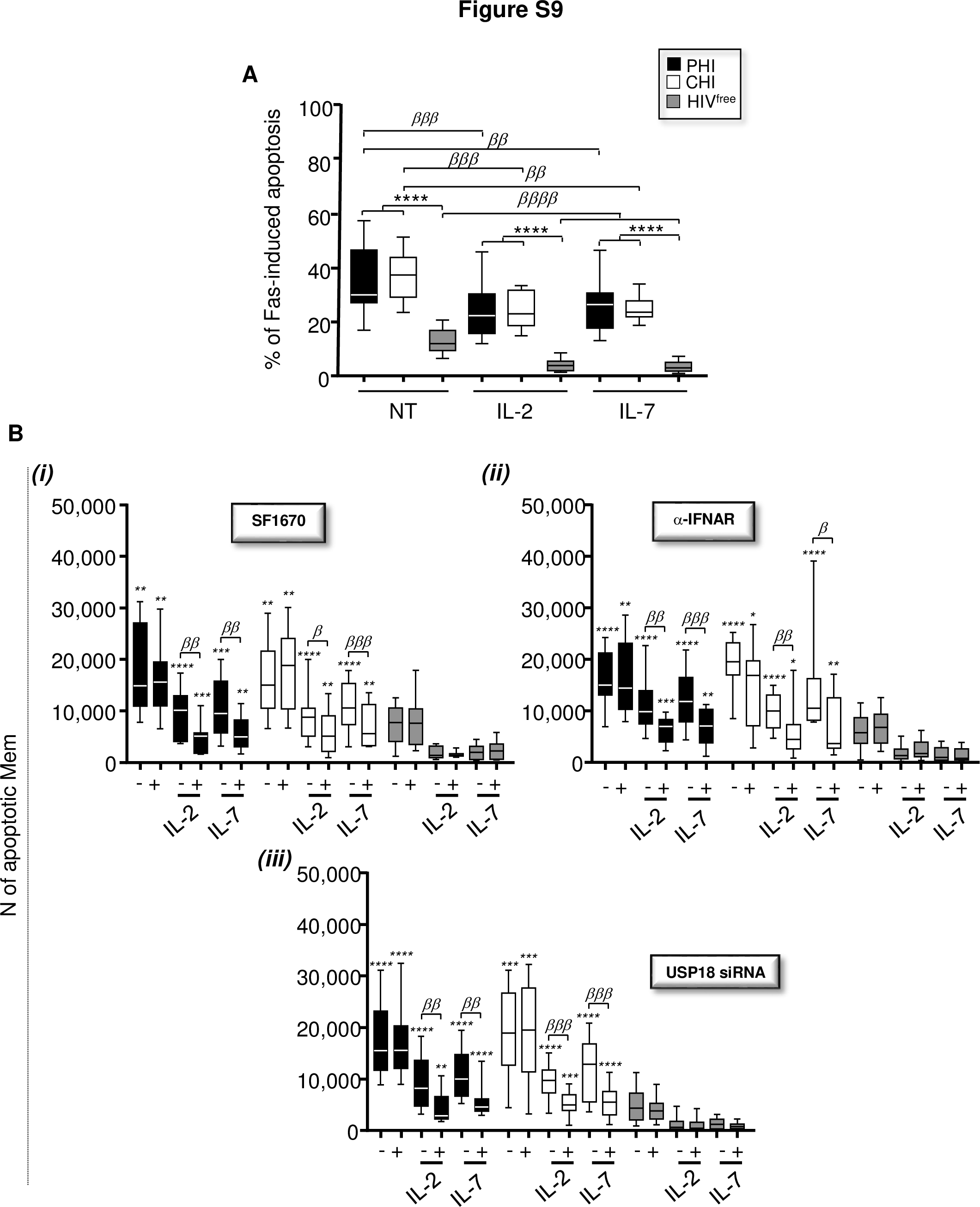

Supplement: S9 Fig — (A) Percentage of Fas-induced apoptosis in Mem in the presence or absence of IL-2 or IL-7 stimulation. Fas-induced apoptosis was calculated according the formula: % of apoptosis in Mem with CH11 –% of apoptosis in Mem without CH11 (n = 10). (B) Number of Fas-induced apoptotic Mem in the presence or absence of IL-2 or IL-7 stimulation in Mem that have been pre-treated for 48h with SF1670 (i), α-IFNAR or its respective isotype control (ii), or pre-transfected or not for 48 hours with USP18 siRNA (iii). Number of Fas-induced apoptototic Mem was calculated according the formula: N of apoptotic Mem with CH11 –Number of apoptotic Mem without CH11 (n = 10). The error bars indicate standard deviations from the means. β, symbol used for paired t test (comparison between treated Mem and control). *, symbol used for Mann-Whitney test (comparison between study groups). (TIF) [file ppat.1008060.s012.tif]

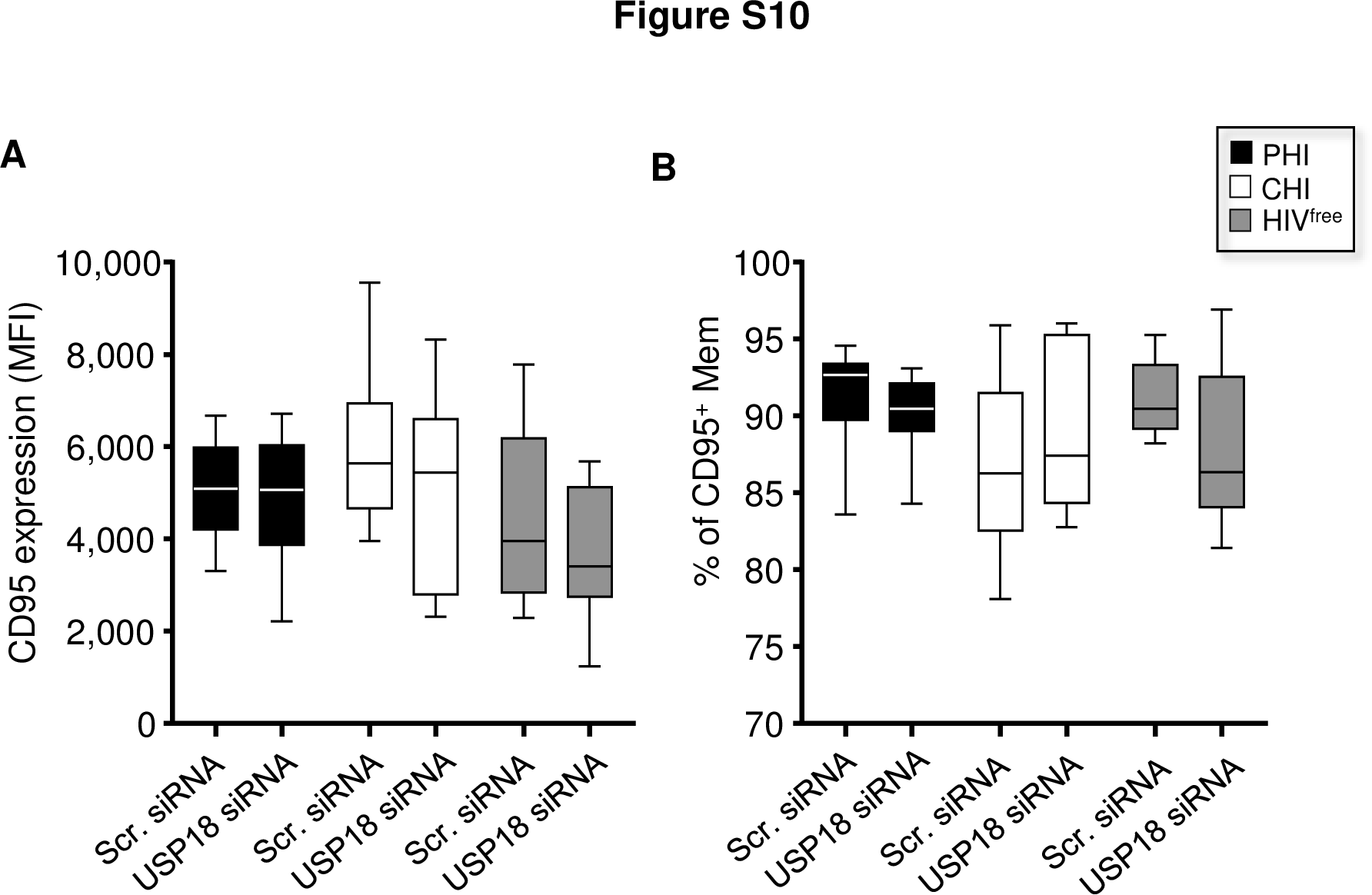

Supplement: S10 Fig — (A,B) Expression levels of CD95 in Mem that have been pre-transfected 48 hours with specific USP18 siRNA or scramble control. Results are expressed as (A) mean fluorescence intensities and (B) percentages of positive cells. (A,B) (n = 10). The error bars indicate standard deviations from the means. (TIF) [file ppat.1008060.s013.tif]

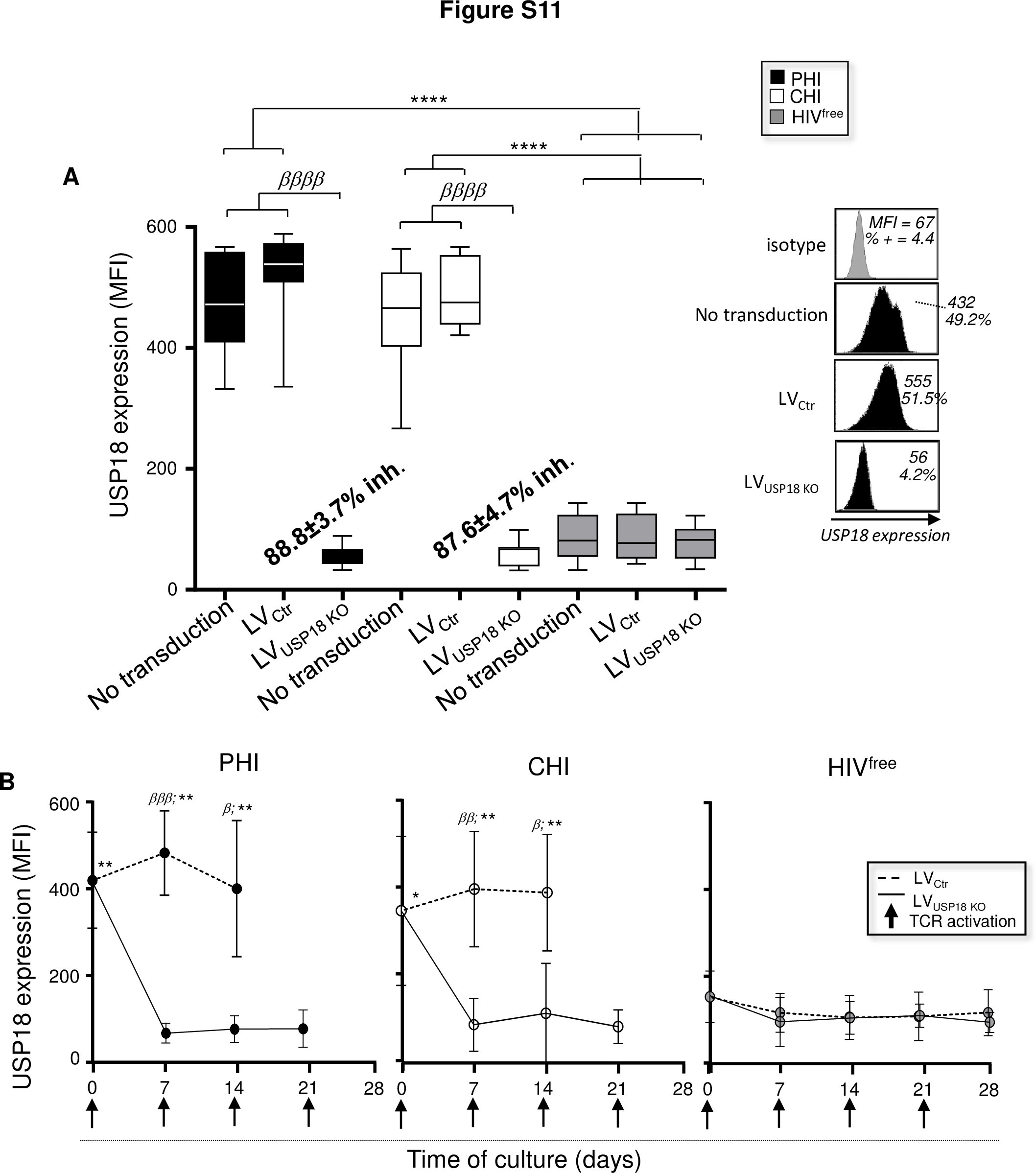

Supplement: S11 Fig — (A) USP18 expression in Mem that have been transduced or not for 48 hours with lentiviral CRISPR/Cas9 vectors mediating USP18 gene editing (lentiviral vectors for USP18 knock-out or LVUSP18 KO) or control lentiviral vectors (LVCtr) in PHI, CHI and HIVfree subjects (MFI; n = 10). Representative histograms including isotype control are also shown on the right side for one PHI subjects. (B) USP18 expression in Mem from PHI, CHI and HIVfree subjects following Mem TcR activation every 7 days for 28 days in the presence or absence of CRISPR/Cas9 mediated USP18 gene editing (n = 6). The error bars indicate standard deviations from the means. β, symbol used for paired t test (comparison between treated Mem and control). *, symbol used for Mann-Whitney test (comparison between study groups). (TIF) [file ppat.1008060.s014.tif]

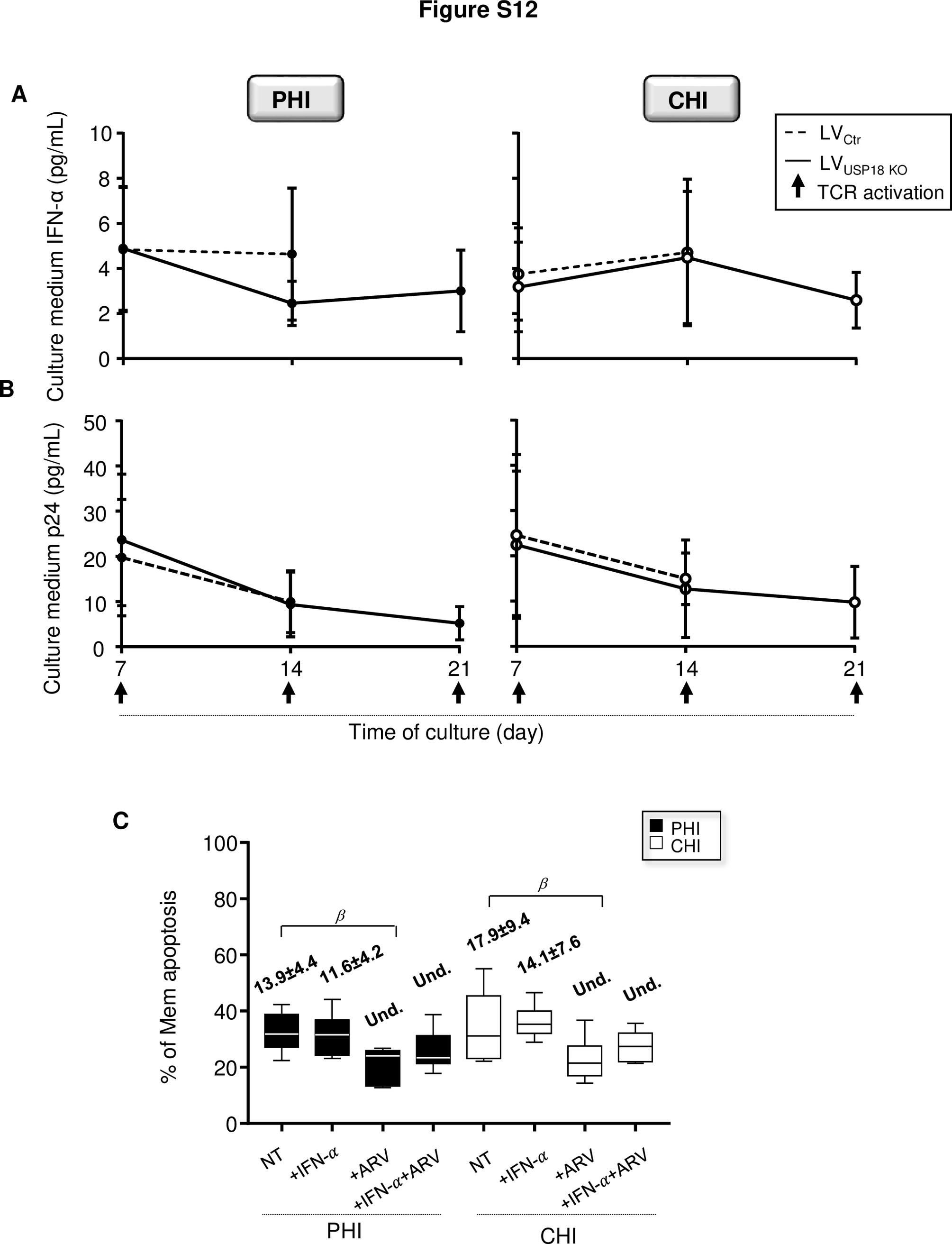

Supplement: S12 Fig — (A) IFN-α and (B) p24 levels in culture medium from PHI and CHI subjects at day 7, 14 and 21 days of culture when Mem have been transduced or not at day 0 with LVUSP18 KO. Results are expressed in pg/mL (n = 6). (C) Apoptosis levels in Mem from PHI and CHI at day 7 of culture when treated or not with two antiretrovirals (ARV) (n = 6). In this context, we used 10 μM AZT and one fusion inhibitor to prevent any de novo infection (100 nM T20). We also added or not at day 0 of cultures 150 IU/ml IFN-α to sustain IFN-I signaling in the absence of virus. The levels of HIV-1 p24 in pg/ml assessed at day 7 in supernatants are also indicated in bold for all conditions (Und., undetectable levels). The error bars indicate standard deviations from the means. β, symbol used for paired t test (comparison with no treatment or NT). (TIF) [file ppat.1008060.s015.tif]

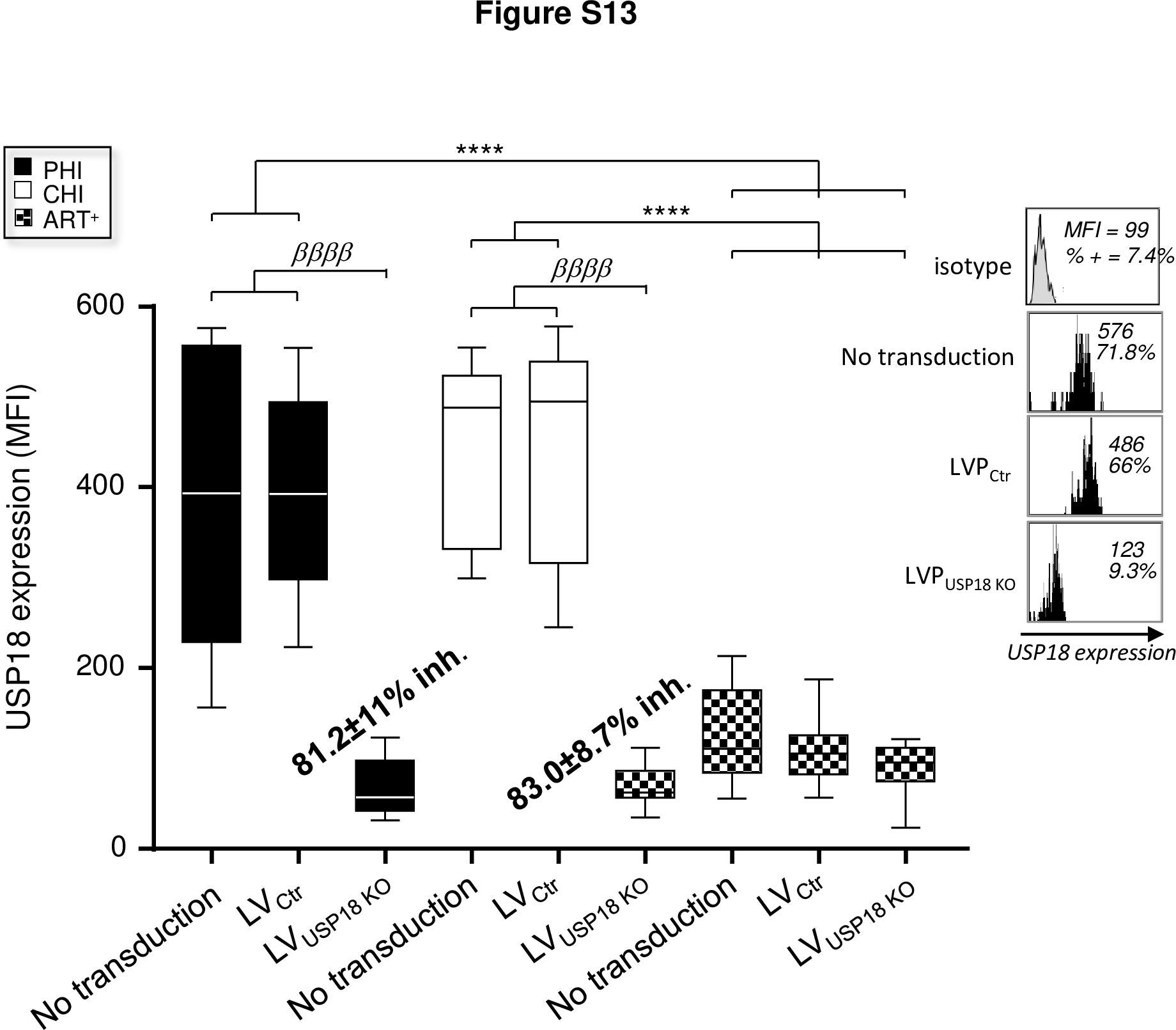

Supplement: S13 Fig — USP18 expression levels (MFI) in IFNγ+ HIV-1-specific CD4 T-cells following 18 hours of Gag stimulation when cells have been pre-transduced or not with LVUSP18 KO. Representative histograms including isotype control are also shown on the right side for one PHI. (n = 10). The error bars indicate standard deviations from the means. β, symbol used for paired t test (comparison between treated Mem and control). *, symbol used for Mann-Whitney test (comparison between study groups). (TIF) [file ppat.1008060.s016.tif]

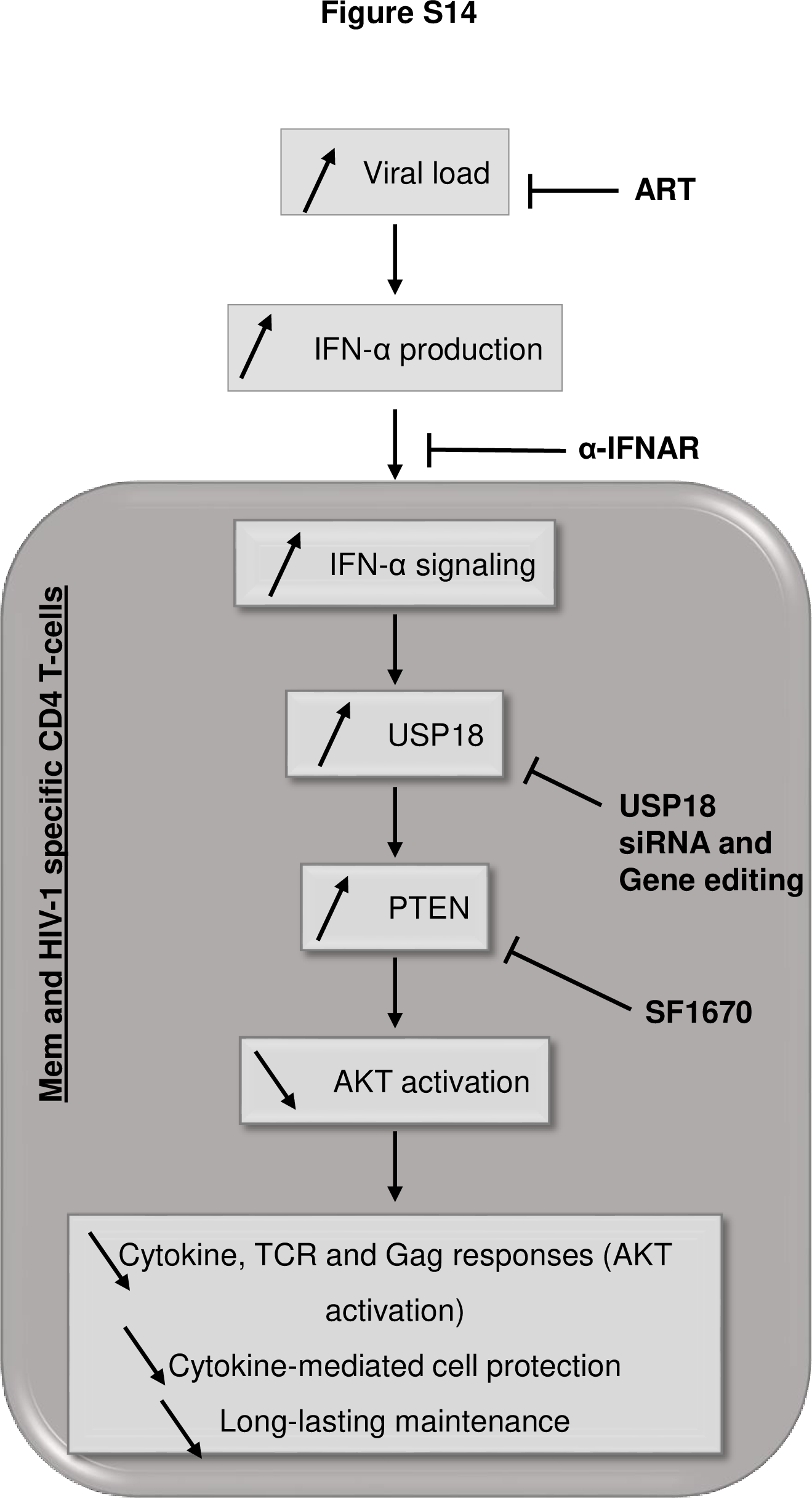

Supplement: S14 Fig — (TIF) [file ppat.1008060.s017.tif]

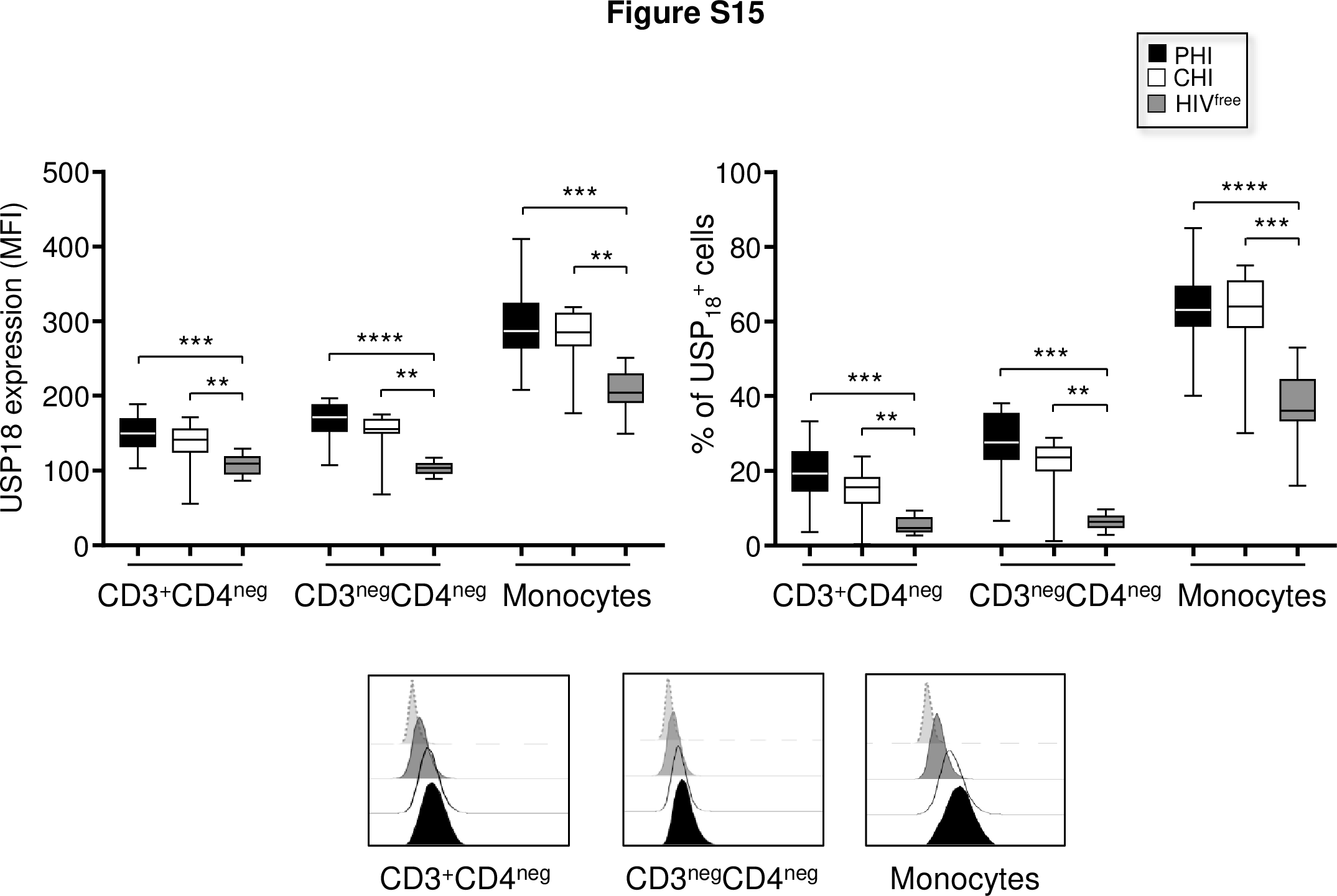

Supplement: S15 Fig — Constitutive expression of UPS18 is higher in CD3+CD4neg, CD3negCD4neg and monocytes from PHI and CHI when compared to HIVfree subjects as determined with MFI values (left) and percentages of positive cells (right) (n = 10). Representative histograms including isotype control are also shown below. The error bars indicate standard deviations from the means. *, symbol used for Mann-Whitney test (comparison between study groups). (TIF) [file ppat.1008060.s018.tif]

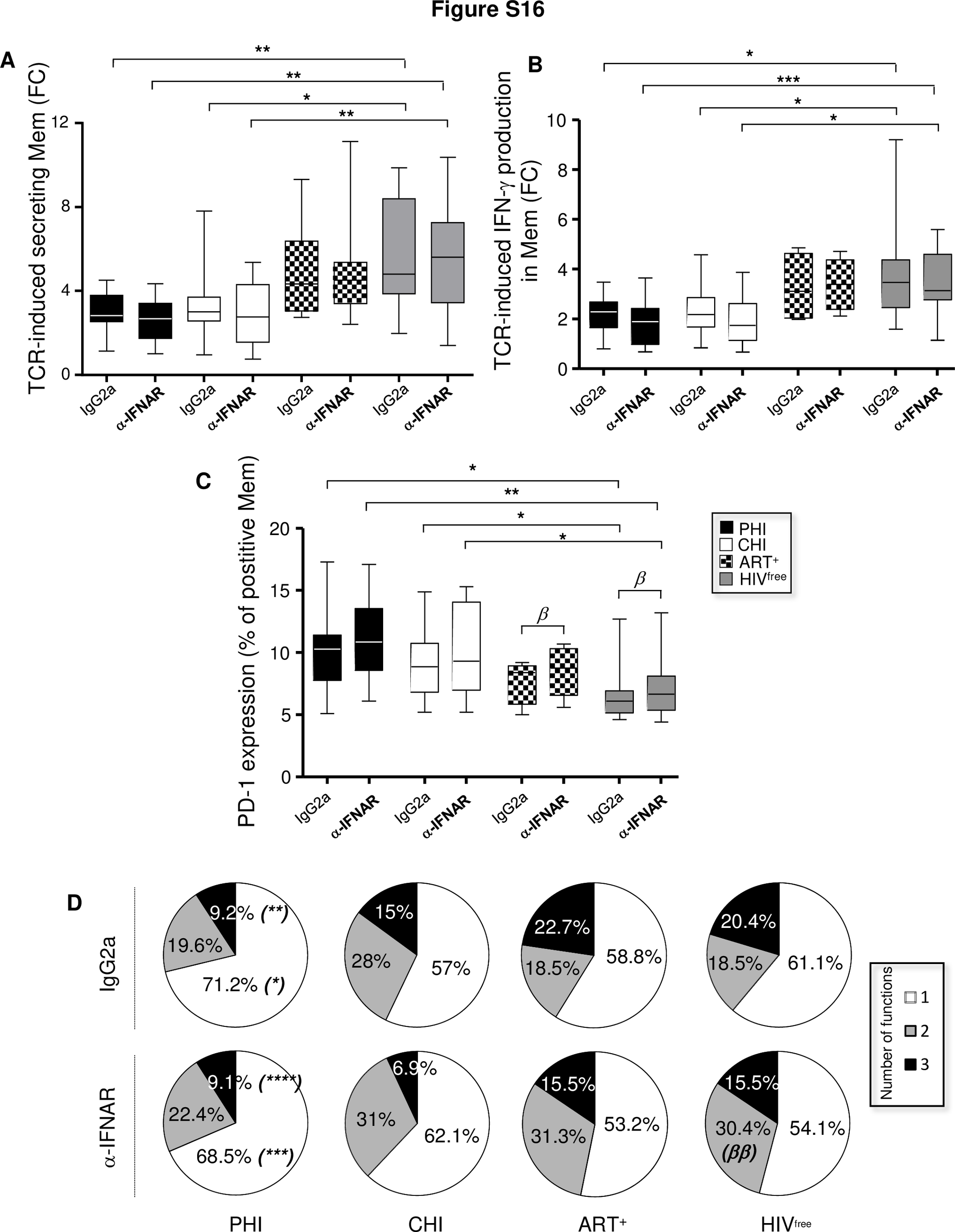

Supplement: S16 Fig — (A) Results are shown as the increases in FC (Fold change) of TCR-induced secreting Mem in the presence or absence of IFNAR blockade. Secreting cells are defined as producing either IFN-γ, TNF-α, IL-2, or combinations of multiple of them. No statistical differences were observed in the percentage of secreting Mem in the absence of TCR activation for all study groups of subjects. (B) Results shown are the increases in FC of TCR-induced IFN-γ producing Mem in the presence or absence of IFNAR blockade. (C) Percentages of PD-1 positive cells in Mem in the presence or absence of IFNAR blockade. (D) Representative distribution of secreting PD-1 positive Mem in the presence or absence of IFNAR blockade. (A-D) N = 10. The error bars indicate standard deviations from the means. β, symbol used for paired t test (comparison between Mem treated α-IFNAR and IgG2a controls). *, symbol used for Mann-Whitney test (comparison with HIVfree controls). (TIF) [file ppat.1008060.s019.tif]

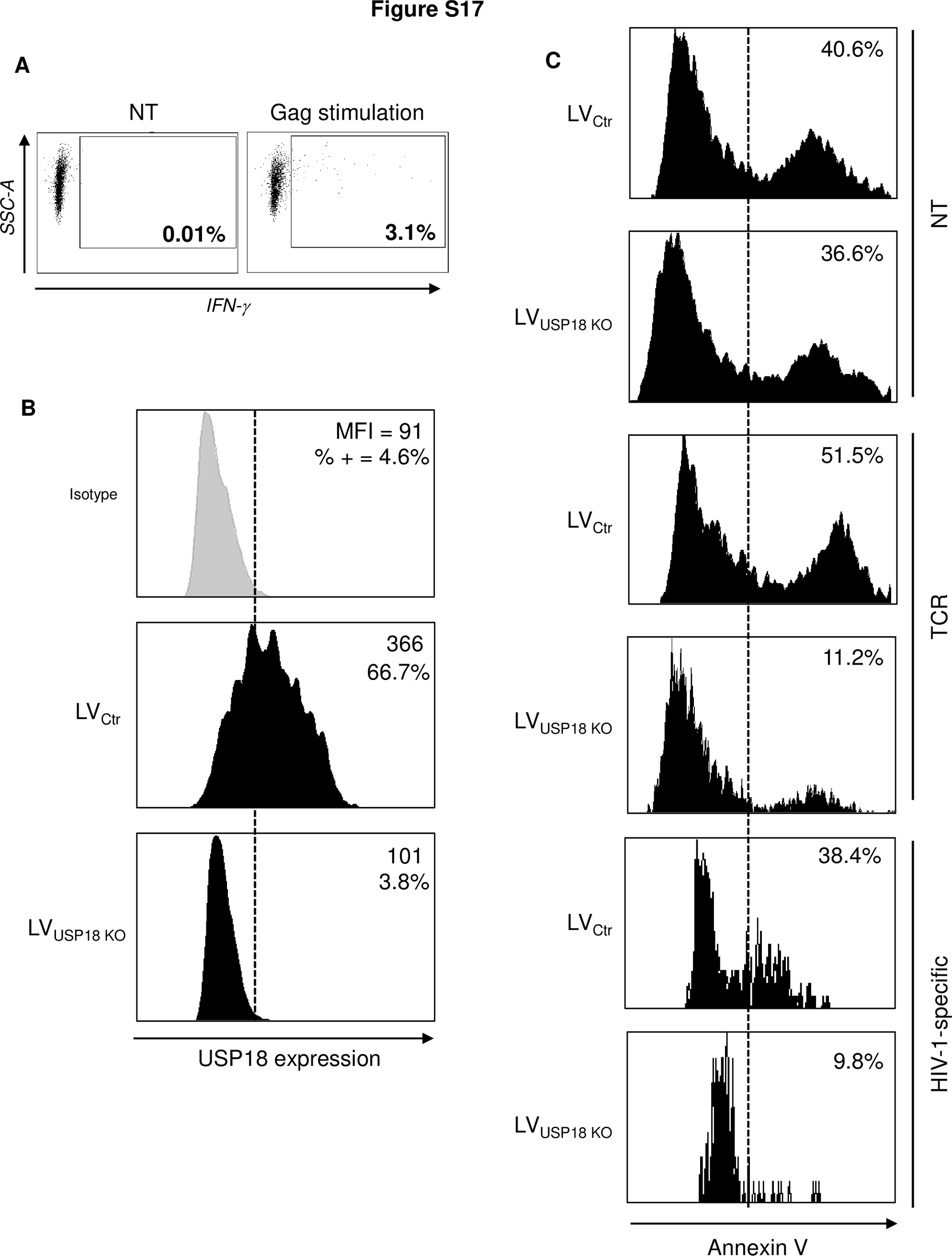

Supplement: S17 Fig — Briefly, spleen cells were collected and transduced for 48 hours with LVCtr or LVUSP18 KO. Transduced cells were then activated or not using anti-CD3 and anti-CD28 Abs (TCR stimulation), or p55 Gag and anti-CD28 Abs (HIV stimulation). (A) Gating strategy to detect the virus-specific CD4 T-cells at 18 hours of HIV-1 stimulation using IFN-γ expression. (B) USP18 expression in gated Mem after 48 hours of cell transduction. Isotype control is also shown in grey. (C) Levels of apoptosis on transduced CD4 T-cells after cell activation. Representative histograms show the apoptosis in Mem and IFN-γ+ virus-specific cells for TCR and HIV stimulation, respectively. (TIF) [file ppat.1008060.s020.tif]
